# The Influence of C–O–C and C–H Functionalities on Competitive Adsorption of Dimethyl Ether and Propane on FAU-Type X Zeolites

**DOI:** 10.1021/acsomega.6c06435

**Published:** 2026-09-04

**Authors:** Malte Menk, Christian Bläker, Christoph Pasel, Dieter Bathen

**Affiliations:** † Thermal Process Engineering, 120335 University of Duisburg-Essen, Lotharstraße 1, Duisburg D-47057, Germany; ‡ Institute for Environment & Energy, Technology & Analytics e. V. (IUTA), Bliersheimer Straße 58-60, Duisburg D-47229, Germany

## Abstract

This study investigates the influence of ether (C–O–C) and aliphatic (C–H) functionalities on competitive adsorption in porous materials. Therefore, comparative isothermal adsorption experiments with dimethyl ether (DME) and propane (C_3_H_8_) were performed on FAU-type X zeolites (NaX and CaNaX) between −20 and 25 °C. Breakthrough curves measured by FTIR on fixed beds were used to derive both pure-component and binary mixture isotherms. Additionally, mixture equilibria were calculated using the Ideal Adsorbed Solution Theory (IAST). Due to its permanent dipole, DME exhibits significantly stronger cation–dipole interactions compared to nonpolar propane, resulting in higher loadings, especially at low concentrations. This effect is more significant on CaNaX, where divalent Ca^2+^ ions provide stronger electrostatic interaction sites. In contrast, propane adsorption is governed primarily by weaker dispersion and induced-dipole interactions, leading to lower affinities and capacities under the same conditions. In binary adsorption, a pronounced selectivity toward DME is observed for both zeolites across the entire temperature range. DME largely reaches its pure-component loadings, while propane is almost completely displaced. However, deviations occur on CaNaX at lower temperatures, where kinetic limitations prevent complete displacement of propane, resulting in residual propane loadings and reduced DME capacities. Mixture isotherms calculated using IAST qualitatively capture the strong selectivity and competitive behavior but show quantitative deviations from the experimental data, particularly at low loadings.

## Graphical abstract

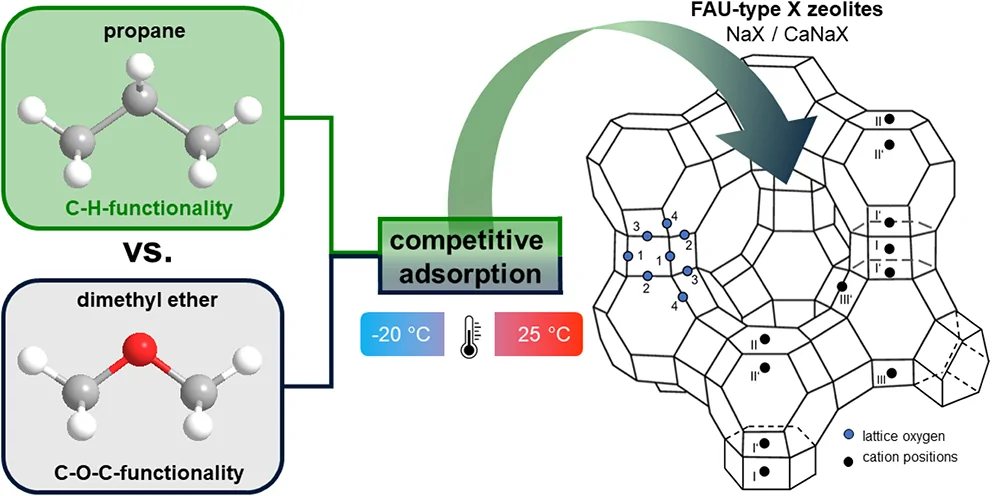

## 1 Introduction and Theoretical Background

Oxygen-containing volatile organic compounds (VOCs) such as ethers play a central role in the chemical industry, whether as solvents, blowing agents or as building blocks for pharmaceuticals and anesthetics.^1–3^ An example is the inhalation anesthetic sevoflurane, which features an ether bridge (C–O–C) alongside a fluorinated carbon chain. While these functional groups provide the desired physicochemical properties for medical use, the emission of such substances into the atmosphere has an environmental impact due to their persistence and global warming potential. The removal and recovery of these highly functionalized molecules from exhaust air streams has therefore gained technical relevance.^4–7^ One option is adsorptive separation using porous materials such as zeolites.^8–10^ In particular, Faujasite (FAU) type X zeolites, such as zeolite NaX and the Ca^2+^-exchanged zeolite CaNaX, are used due to their comparatively high adsorption capacity, adjustable surface polarity and selective interactions in gas mixtures.^11–13^ However, the adsorption of multicomponent mixtures poses a challenge, as complex displacement mechanisms may occur.^14–17^ To understand the adsorption of complex molecules such as sevoflurane on a mechanistic level, it is necessary to examine the influence of individual functional groups. In a previous study, the influence of the C–F bond compared to the C–H bond was analyzed in detail using the propane/perfluoropropane model system.^18^ Since sevoflurane is a fluorinated ether, this study focuses on a comparison between the ether bridge (C–O–C) and the aliphatic structure (C–C–C). A binary mixture of dimethyl ether (DME) and propane serves as the model system for this purpose. Both molecules have a similar geometry, but differ fundamentally in their polarity.

The adsorption of propane on FAU zeolites has already been described in detail in the literature. Studies by Birkmann et al. and Schmittmann et al. describe the adsorption behavior on NaX primarily as a combination of dispersion forces and induced dipole interactions with the lattice cations. In this context, attractive lateral interactions lead to characteristic S-shaped isotherms with increasing surface coverage.^19,20^ Bläker et al. demonstrated that the adsorption enthalpy depends on the charge of cations. The exchange for divalent Ca^2+^ cations leads to significantly higher binding energies compared to Na^+^ due to the higher charge density.^21^

In contrast, data on the adsorption of DME on FAU zeolites is incomplete. Lad et al. investigated DME adsorption on 4A and 5A zeolites using isothermal and calorimetric analyses. They demonstrated that pore size and accessible surface area are the dominant parameters. Due to larger pores and a higher surface area, zeolite 5A exhibits an 8-fold higher adsorption capacity, with adsorption being controlled by intracrystalline diffusion, whereas in zeolite 4A it is surface-limited by steric effects. Moderate adsorption enthalpies (20–26 kJ/mol) indicate a physisorptive adsorption mechanism.^22^ Furthermore, Nie et al. demonstrated in FAU zeolites that a high surface area, large pores and a low Si/Al ratio in particular enhance adsorption by strengthening electrostatic interactions with the polar DME.^23^

Although the pure component adsorption has been investigated, no data are currently available on the adsorption of mixtures of propane and DME on FAU zeolites. To fill this data gap, the present study systematically investigates the adsorption of propane/DME mixtures on zeolites NaX and CaNaX at three temperatures (−20 °C, 0 °C, 25 °C). Both pure component and mixture isotherms were measured, mixture isotherms are modeled using IAST. The aim is to gain a mechanistic understanding of the role of C–O–C- functionality in adsorption process.

## 2 Experimental Section

### 2.1 Materials

FAU-type X zeolites were used as adsorbents, analogous to our previous study.^18^ The topology of these zeolites is built by the connection of sodalite cages (β-cages) using double six-membered rings (D6R), forming large supercages (diameter ≈ 1.37 nm). These supercages are accessible through 12-membered ring apertures (diameter ≈ 0.74 nm). Topology and structural properties are well documented in literature.^24–28^ Due to the isomorphous substitution of Si^4+^ by Al^3+^, negative framework charges arise, which are compensated by extra-framework cations located at defined crystallographic positions (sites I, I′, II, II′, III and III′). Only cations at sites II, III, and III′ are accessible to molecules used in this study (see Figure 1).

**Figure 1:**
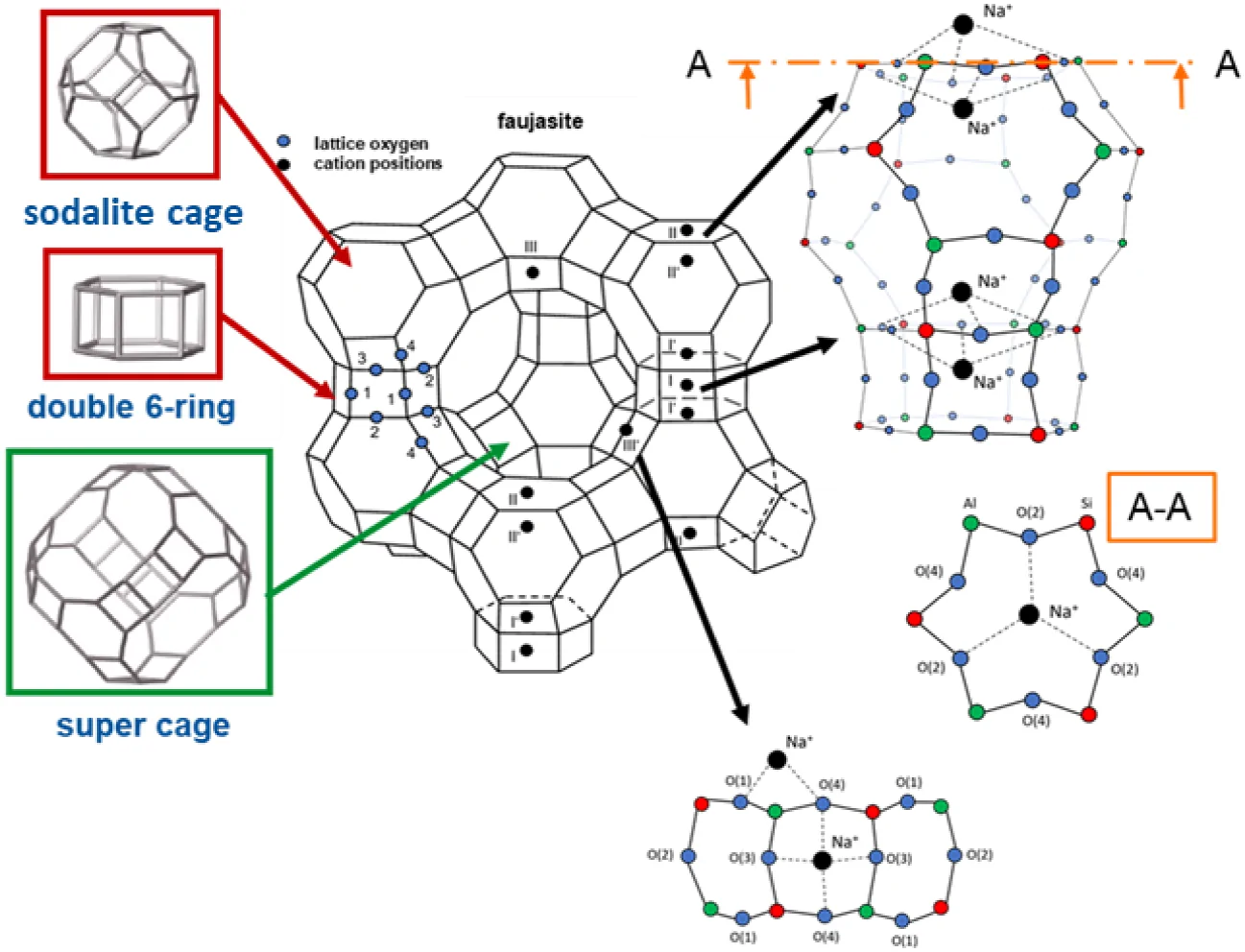
Schematic representation of sodalite cage, double six-membered ring, supercage, and FAU-type X zeolite topology with crystallographic cation positions, adapted from ref 13.

Two FAU-type X zeolites with a Si/Al ratio of 1.175 were used. Zeolite NaX contains exclusively sodium cations (Na^+^), whereas in zeolite CaNaX, 80% of Na^+^ ions are replaced by Ca^2+^ ions. During ion exchange, two monovalent Na^+^ are substituted by one divalent Ca^2+^, resulting in a reduced total cation number per unit cell from 88 in zeolite NaX to 52 in zeolite CaNaX and a redistribution of cations among sites II and III/III′. The presence of Ca^2+^ increases the local electrostatic field strength and thus enables stronger interactions with polar adsorptives such as DME. Both materials were synthesized by Chemiewerke Bad Köstritz GmbH. The bulk elemental compositions of both zeolites were determined from XRF data provided by Chemiewerke Bad Köstritz GmbH. The relevant oxide contents and the derived Si/Al ratios, cation numbers per unit cell, and Ca^2+^ exchange degrees are summarized in Table 1.

**Table 1:** Chemical Properties of Zeolite Materials (Determined by XRF (X-ray Fluorescence Spectroscopy))

		Chemical Composition (wt %)					
Zeolite	Si/Al Ratio	Al_2_O_3_	SiO_2_	Na_2_O	CaO	Ca^2+^exchange degree (%)	Abbreviation
Na_88_Si_104_Al_88_O_384_	1.174	26.70	36.90	17.70	0.0325	–	NaX
Na_16_Ca_36_Si_104_Al_88_O_384_	1.174	26.30	36.40	3.22	12.50	80	CaNaX

PXRD measurements were performed by Max-Planck-Institut für Kohlenforschung (Mülheim, Germany) to determine the distribution of Na^+^ and Ca^2+^ ions across the crystallographic cation sites and to verify that the FAU framework remained intact after ion exchange. XRPD data were collected on a Rigaku SmartLab diffractometer (Rigaku Ltd., Japan). The data were recorded with a HyPix-3000 detector (Rigaku Ltd., Japan) system. In addition, an XRK900 reaction chamber (Anton Paar Group, Graz, Austria) was installed. The XRPDs were analyzed with the “DiffracPlus Topas 6” software (Bruker AXS GmbH, Karlsruhe, Germany). The exact framework structures of the zeolite FAU type X samples were obtained using the cubic space group Fm3 ®m. The cation positions were determined via the charge flipping algorithm based on XRF analysis, the final cation positions and the respective amount of Ca^2+^ and Na^+^ were determined by Rietveld refinements. The calculated cation distributions for NaX and CaNaX are given in Table 2.

**Table 2:** Experimentally Determined Cation Distribution of the FAU-Type Zeolites NaX and CaNaX, Based on Rietveld Refinement of PXRD Data

		Site I/I′		Site II		Site III/III′	
Zeolite	Ca^2+^ exchange degree (%)	Na^+^	Ca^2+^	Na^+^	Ca^2+^	Na^+^	Ca^2+^
NaX	0	32	—	32	—	24	—
CaNaX	80	5	13	9	22	2	1

The structural parameters obtained from argon adsorption at 87 K are comparable for both materials. The BET surface areas, determined according to DIN ISO 9277,^29^ are 764.2 m^2^ g^–1^ for zeolite NaX and 746.6 m^2^ g^–1^ for zeolite CaNaX, while the corresponding pore volumes, calculated using the Gurvich rule in accordance with DIN 66134 ^30^ are 0.2864 cm^3^ g^–1^ and 0.2885 cm^3^ g^–1^.

Propane with a purity >99.5% (Air Liquide) and DME with a purity >99.9% (Linde Gas) were used as adsorptives. Nitrogen from the university supply network, with a purity of >99.999% and a dew point of −80 °C, was used as the inert carrier gas. Table 3 gives the chemical and physical properties of the compounds.

**Table 3:** Chemical and Physical Properties of the Adsorptives and the N_2_ Carrier Gas Used

Property	Unit	Nitrogen	Propane	Dimethyl ether
Molecular formula	—	N_2_	C_3_H_8_	CH_3_OCH_3_
Molar mass^31^	g mol^–1^	28	44	46
Critical molecular dimension, *d*_min1_	10^–10^ m	3.1	4.0	3.8
Critical molecular dimension, *d*_min2_ ^31,32^	10^–10^ m	3.1	4.5	4.2
Polarizability volume^33,34^	10^–30^ m^3^	1.7	5.9	5.2
Dipole moment^31,35^	10^–30^ C m	0	0.3	4.3

C_3_H_8_ is an alkane with a negligibly small dipole moment, as the electronegativity difference of about 0.3 between the C and H atoms is very small and the molecular geometry is symmetric. Propane thus primarily forms dispersion and induction interactions, both with the adsorbent and with other adsorbed molecules. DME can be regarded as an ether derivative of propane. The C–O bonds exhibit a significantly higher polarity than C–C or C–H bonds due to the large electronegativity difference of ∼0.9 between carbon and oxygen. Consequently, DME possesses a pronounced molecular dipole moment (μ ≈ 4.3 × 10^–30^ C m) and an asymmetric electron distribution, due to its bent C–O–C geometry. This polar character enables the formation of stronger polar interactions in addition to dispersion forces. DME exhibits cation–dipole interactions with the adsorbent and dipole–dipole interactions with other adsorbed DME molecules, whereas dipole–induced dipole interactions are formed with adsorbed propane molecules. Furthermore, there are interactions with the polar oxygen atoms in the framework. Since the C–O–C functionality simultaneously changes several molecular properties, the comparison reflects their combined influence, with molecular polarity and cation–dipole interactions expected to be particularly important. In the following discussion, in case of DME, we focus on the cation–dipole interactions, as they are the strongest forces which dominate the adsorption.

### 2.2 Experimental Setup and Procedure

Following the procedure described in our previous publication,^18^ breakthrough curves were recorded using a fixed-bed adsorber (length ≈ 180 mm, inner diameter ≈ 13 mm; see Figure 2). The column was equipped with a double jacket for precise temperature control by means of liquid media. Additionally, the pipes upstream of the adsorber were thermostated over a length of 1.5 m. This configuration enabled accurate control of the adsorption temperature within the range of −20 to 25 °C.

**Figure 2:**
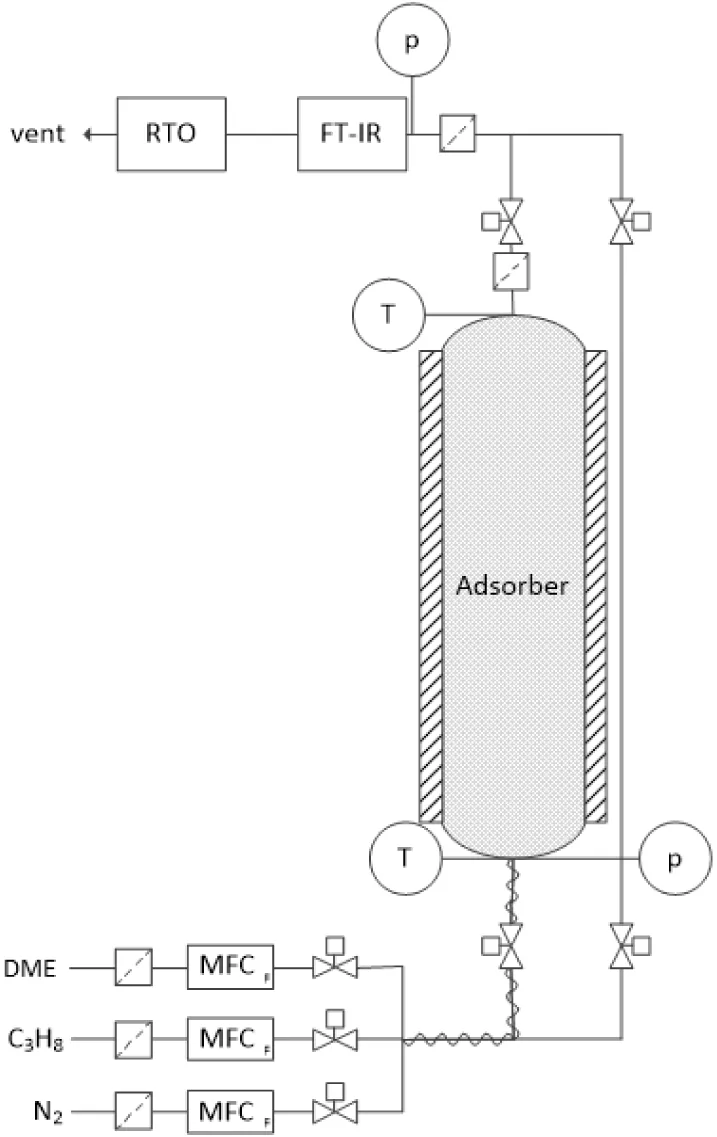
Schematic setup of the fixed bed adsorber. “T” indicates the position of a thermocouple, “p” indicates the position of a pressure sensor, “MFC” denotes a thermal mass flow controller, “FT-IR” denotes the Fourier-transform infrared spectrometer and “RTO” denotes the regenerative thermal oxidizer.

For quantitative determination of gas concentrations, a Fourier-transform infrared spectrometer (FT-IR, Bruker Matrix-MG2) was employed, as described previously.^18^ The optical setup comprises a silicon carbide light source, a thermoelectrically cooled mercury cadmium telluride (MCT) detector, and a multireflection gas cell (path length = 2 m) with ZnSe windows. The system allows simultaneous quantification of multiple components even at low concentrations.

Prior to each experiment, the zeolites were conditioned under an inert N_2_ atmosphere at 300 °C for 4 h to remove adsorbed species and residual moisture (final water content ≈ 1.1 wt %). Temperature equilibration was achieved by continuous nitrogen purging until the target temperature was reached. Gas concentrations were adjusted using thermal mass flow controllers (MFCs). All experiments were conducted at an absolute pressure of 1 bar. The difference between the total pressure and the sum of the partial pressures of propane and DME corresponds to the partial pressure of the carrier gas, nitrogen. Before adsorption, the gas mixture was passed through a bypass until a stable adsorptive concentration was confirmed by FT-IR. Adsorption was initiated by directing the gas mixture into the adsorber.

Equilibrium was assumed when the adsorptive concentrations at the inlet and outlet were identical, with a concentration change of less than 20 ppm over at least 15 min as the equilibrium criterion. Pure-component isotherms were measured cumulatively by stepwise increasing the adsorptive concentration after equilibrium was reached.

Binary mixture experiments were performed under two conditions: (i) equimolar mixtures at different total concentrations and (ii) mixtures with constant total concentration but varying mole fraction, analogous to our methodology described in ref 18. In the latter case, the propane concentration was gradually decreased while the concentration of DME was increased accordingly, until only DME remained in the feed gas. Representative concentration profiles are provided in the SI file (S1).

### 2.3 Evaluation of the Experimental Data

In the cumulative experiments, the equilibrium loading *X*_*eq,i*_ in *i*-th step was determined by adding the change in loading during the prevailing step to the previous equilibrium loading $X_{eq_{i-1}}$ , eq 1. The change in loading in *i*-th step was calculated from a mass balance around the adsorber, using the molar inlet flow ṅ*_in,i_*, normalized by the adsorbent mass *m*_*ads*_ and the time-summed normalized concentration differences $\frac{y_{in_{j}}-y_{out_{j}}}{1-y_{out_{j}}}$ at each measurement point *j*. The inlet concentration $y_{in_{j}}$ was determined as the 15 min average of outlet concentration after reaching equilibrium. Integration was performed by summing these terms over all time intervals until equilibrium $j_{i,eq.}$ , multiplying each by the corresponding interval time Δ*t_j_* (approximately 20 s).

1 $$X_{eq_{i}}=X_{eq_{i-1}}+\frac{{ṅ}_{in,i}}{m_{ads}}\times \overset{j_{i,eq.}}{\underset{j_{i,0}}{\sum }}\left[\frac{y_{in_{j}}-y_{out_{j}}}{1-y_{out_{j}}}\right]\times {\Delta t}_{j}$$

In binary adsorption, the presence of a second component with mole fraction $y_{n_{i,2}}$ was considered, extending eq 1 to eq 2. A detailed derivation of the mass balances is provided in the SI file (S2, S3).

2 $$X_{eq_{i,1}}=X_{eq_{i-1,1}}+\frac{{ṅ}_{in,i}}{m_{ads}}\times \overset{j_{i,eq.}}{\underset{j_{i,0}}{\sum }}\left[y_{in_{i,1}}-y_{out_{j,1}}\left[\frac{1-y_{in_{i,1}}-y_{in_{i,2}}}{1-y_{out_{j,1}}-y_{out_{j,2}}}\right]\right]\times {\Delta t}_{j}$$

To compare the loadings with the number of cations and thus analyze the adsorption mechanisms, the loadings (mol kg^–1^) were converted to molecules per unit cell using eq 3. Here *N*_*A*_ denotes Avogadro’s constant and *m*_*uc*_ the mass of a unit cell.

3 $$X\left[\frac{molecules}{uc}\right]=X\left[\frac{mol}{kg}\right]\cdot N_{A}\left[\frac{molecules}{mol}\right]\cdot m_{uc}\left[\frac{kg}{uc}\right]$$

The global temperature-dependent Sips isotherm (eqs 4 to 7) was used to fit experimental adsorption data at various temperatures. Here, *X_mon_* (*T*) denotes the saturation capacity, *b*(*T*) the affinity constant and *n*(*T*) the heterogeneity constant, which are all described as temperature-dependent functions. The corresponding reference values *X_mon_* (*T_Ref_*), *b*_0_(*T_Ref_*) and *n*_0_(*T_Ref_*) represent the respective parameters at a chosen reference temperature *T_Ref_* of −20 °C. The parameters χ, Q, α are fitting constants that govern the temperature dependence of the respective functions. The reference isotherm parameters *X_mon_* (*T_Ref_*), *b*_0_(*T_Ref_*) and *n*_0_(*T_Ref_*) as well as the temperature-dependence parameters χ, Q and α were fitted globally including all pure-component isotherms using nonlinear regression with the Levenberg–Marquardt algorithm by minimizing the error squares.

4.1 $$X_{eq}\left[T,p_{i,eq}\right]=X_{mon}\left(T\right)\cdot \frac{{\left(b\left(T\right)\cdot p_{i,eq}\right)}^{1/n\left(T\right)}}{1+{\left(b\left(T\right)\cdot p_{i,eq}\right)}^{1/n\left(T\right)}}$$

4.2 $$X_{mon}\left(T\right)=X_{mon}\left(T_{Ref}\right)\cdot \exp\left[\chi \cdot \left(1-\frac{T}{T_{Ref}}\right)\right]$$

4.3 $$b\left(T\right)=b_{0}\left(T_{Ref}\right)\cdot \exp\left[\frac{Q}{RT_{Ref}}\cdot \left(\frac{T_{Ref}}{T}-1\right)\right]$$

4.4 $$\frac{1}{n\left(T\right)}=\frac{1}{n_{0}\left(T_{Ref}\right)}+\alpha \left(1-\frac{T_{Ref}}{T}\right)$$

To quantify the preferential adsorption of DME over propane in the binary mixtures, the adsorption selectivity was calculated according to

5 $$S=\frac{X_{DME}/X_{propane}}{p_{DME}/p_{propane}}$$

where *X*_*i*_ denotes the equilibrium loading and *p*_*i*_ the corresponding partial pressure of adsorptive *i*. For the equimolar gas-phase compositions investigated in this study, the selectivity simplifies to *S*_DME/propane_ = *X*_DME_/*X*_propane_. Analogous, this simplification applies to mixture measurements with a constant total concentration when the molar fraction of both adsorptives in the gas phase is equal. Values greater than unity indicate preferential adsorption of DME over propane.

IAST, developed by Myers and Prausnitz,^36^ was applied to predict the adsorption equilibrium of binary mixtures. IAST was selected as it allows the prediction of mixture adsorption equilibria from the pure-component isotherms without introducing additional mixture-specific fitting parameters or additional assumptions. Analyzing the deviations between IAST predictions and experimental adsorption data for mixtures makes it possible to distinguish mixture effects from the interactions of the pure components with the adsorbent. In this study we used the following eq 9:

6 $$\Pi =\frac{\pi \times A}{R\times T}=\int_{0}^{p_{i}^{0}\left(T,\pi \right)}\frac{X_{eq,i}}{p_{i}^{0}}\;dp_{i}^{0}$$

Here, Π denotes the reduced spreading pressure, π the spreading pressure, *A* the specific surface area of the adsorbent, $p_{i}^{0}\left(T,\pi \right)$ the hypothetical vapor pressure of the pure component *i* at spreading pressure in the adsorbed phase and *X_eq,i_* the loading in adsorption of the pure component. A detailed description of IAST is provided in literature.^36,37^

### 2.4 Experimental Uncertainties

The experimental equilibrium loadings are subject to measurement uncertainties due to variations in molar flow rates, adsorbent masses, and inlet/outlet adsorbate concentrations. FT-IR concentration measurements have a statistical uncertainty of ±1.5%, MFCs ± 0.04–0.2%, and the adsorbent balance ± 0.03 mg. Using Gaussian error propagation, the total uncertainty in the equilibrium loading is calculated at 1.9%.

Reproducibility was evaluated through replicate measurements, yielding an error of 5%, which is within an acceptable range but approximately 2.5 times higher than the calculated uncertainty using Gaussian error propagation. Detailed error analysis and device-specific uncertainties are provided in the SI (S4).

## 3 Results and Discussion

The data of the pure component experiments are presented as isotherm plots, with the loading expressed in molecules per unit cell (mc/uc) as a function of the respective partial pressure (Pa). For the mixture experiments, the presentation is analogous, with loading plotted versus partial pressure of the corresponding component.

### 3.1 Pure-Component Adsorption of Propane and DME on Zeolites NaX and CaNaX

Figure 3 shows pure component adsorption isotherms of DME and propane over a temperature range of 25 °C to −20 °C on zeolites NaX and CaNaX. The experimentally determined equilibrium points (symbols) can be described with high accuracy by global temperature-dependent Sips equation as shown by the solid lines.

**Figure 3:**
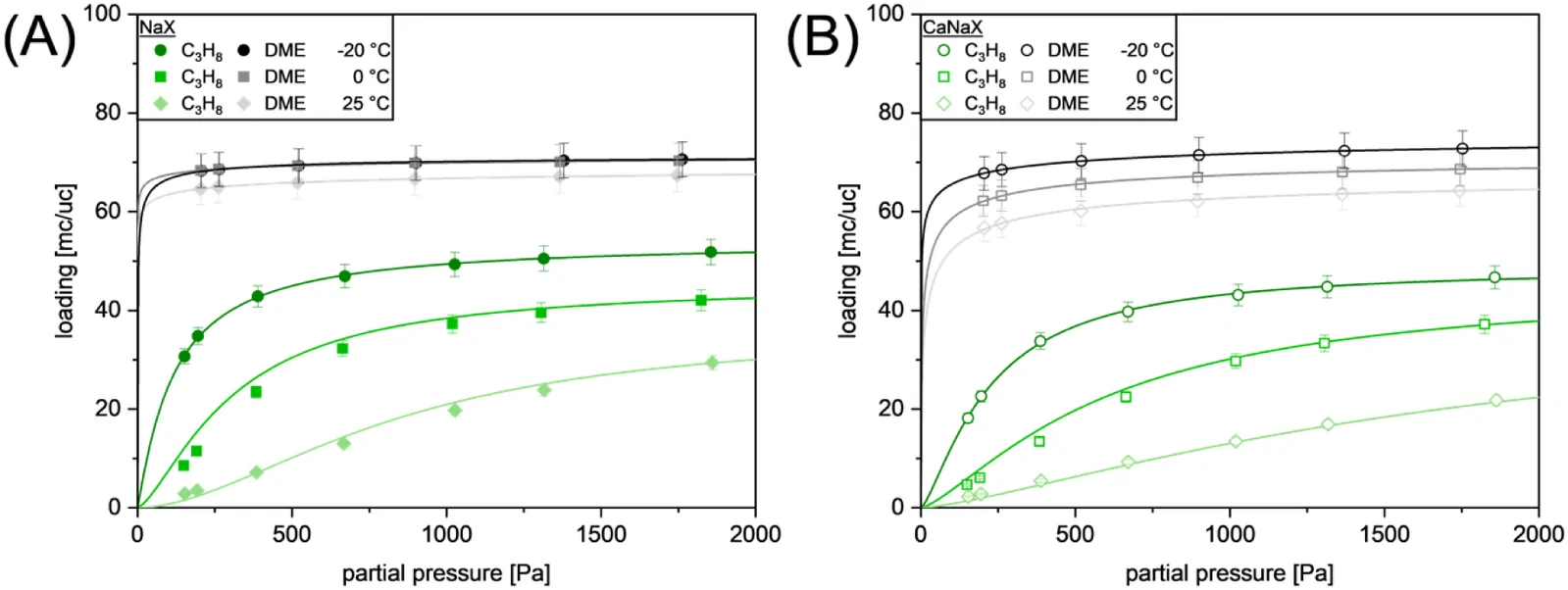
Pure component adsorption isotherms of propane (green) and DME (black) on zeolites NaX (A) and CaNaX (B) at 25 °C (diamonds), 0 °C (squares), and −20 °C (circles). Solid lines: pure component isotherm fit using the Sips equation.

On both zeolites and over the entire range of temperature and concentration DME adsorbs more strongly than propane. Even at low partial pressures below 200 Pa, the DME loading increases sharply as the permanent dipole allows strong cation–dipole interactions with the cations in the zeolite cage. In addition, the DME dipoles interact with the negatively charged lattice oxygen atoms. At 25 °C, DME reaches nearly saturation at the first equilibrium point on both materials. On zeolite NaX the saturation loading is approximately 65 mc/uc (cf. Figure 3A), while on zeolite CaNaX a saturation loading of approximately 60 mc/uc is reached (cf. Figure 3B). In both cases the maximum loading exceeds the number of theoretically available cations. To investigate the adsorption mechanism in further detail, experimental calorimetric adsorption enthalpies were additionally determined using a sensor gas calorimeter. Therefore, a commercially available volumetric measuring device (autosorb iQ3, Quantachrome Instruments, Boynton Beach, FL, USA) was coupled with a sample vessel developed by Bläker et al.^21,38^ A detailed description of the experimental setup, the measurement procedure, and the data analysis is given in the Supporting Information. The load-dependent heats of adsorption are given in Figure 4A and B, with error bars ranging from 1 to 2 kJ/mol. For DME the enthalpies range between 90 and 100 kJ/mol on zeolite NaX and reach a maximum of 130 kJ/mol on zeolite CaNaX. Therefore, they are significantly higher than the adsorption enthalpies of propane (approximately 25–30 kJ/mol). The high values indicate strong physisorption. This confirms the conclusion that, due to its molecular dipole moment, DME can form strong cation–dipole interactions and interact with more than one cation simultaneously. This is attributable to the low Si/Al ratio and the consequently high cation content and leads to a superposition of the potential functions in the cage-like pores. DME exhibits a high affinity toward divalent Ca^2+^ ions in particular while propane can only form weaker induced dipole interactions.

**Figure 4:**
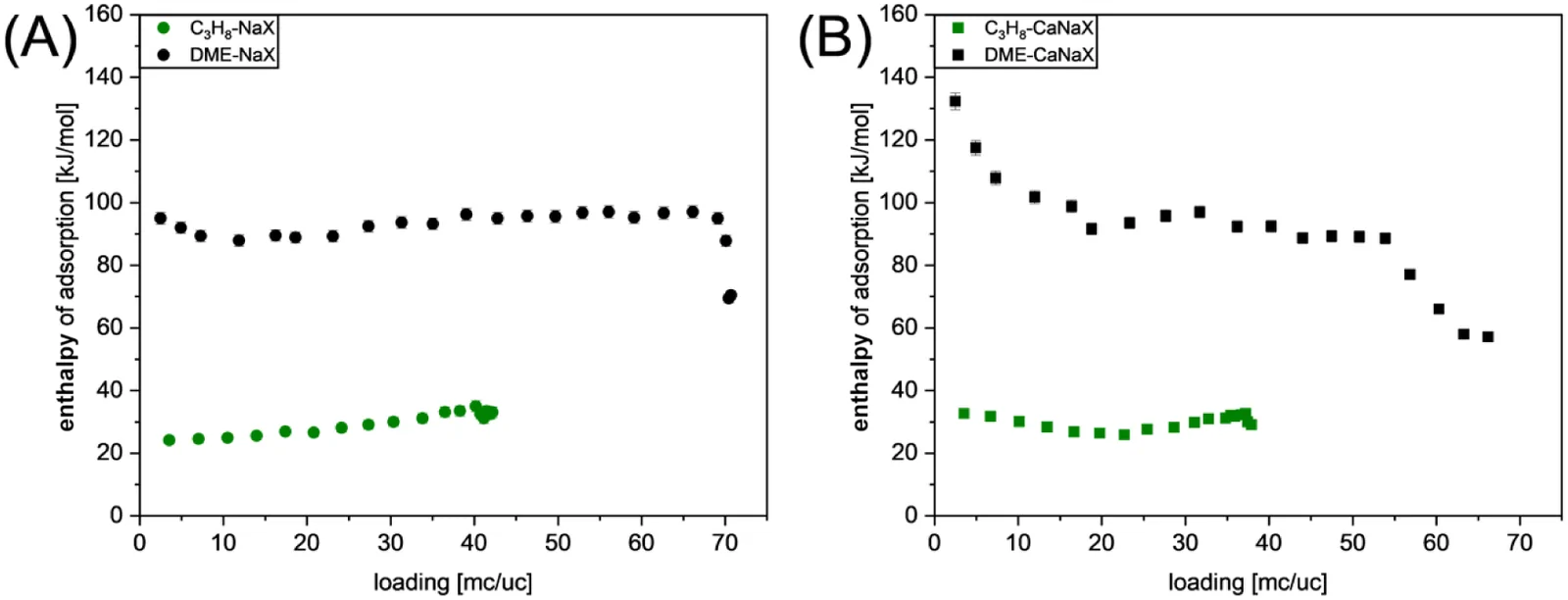
Measured load-dependent enthalpies of adsorption of C_3_H_8_ and DME on NaX (A) as well as C_3_H_8_ and DME on CaNaX (B) at 25 °C.

On zeolite NaX, the enthalpies of propane and DME remain nearly constant and independent of loading over the investigated loading range (see Figure 4A). This suggests that the interaction mechanisms change only slightly in this range. Since the number of adsorbed molecules exceeds the number of available cations, it can be assumed that a single cation can interact simultaneously with multiple adsorptive molecules. Due to the size of the molecules, it can also be expected that an adsorbed molecule can interact simultaneously with multiple cations.

On zeolite CaNaX the adsorption of DME shows a stepwise decrease in enthalpy values as the loading increases (see Figure 4B). From an initial value of 130 kJ/mol, a downward trend is observed reaching a long plateau at 90 kJ/mol. This is followed by a further step down to a level of 60 kJ/mol. At low loadings adsorption may occur preferentially at multiple Ca^2+^ ions. As the loading increases, the mechanism may shift to mixed coordination involving Ca^2+^ and Na^+^ ions. At high loadings only interactions with Na^+^ ions are likely to occur. These assignments represent proposed interpretations rather than proved adsorption structures.

Although DME exhibits significantly higher adsorption enthalpies on zeolite CaNaX than on zeolite NaX, it is remarkable that the initial loading is higher on zeolite NaX. A possible explanation could be that the entropic advantage resulting from higher total number of cations on zeolite NaX outweighs the enthalpic advantage associated with the higher energy value of Ca^2+^ ions. For a more detailed explanation, we refer to our previous publication, in which we have already provided an in-depth discussion of these mechanisms.^18^

Propane exhibits lower loadings on zeolite CaNaX compared to zeolite NaX, since the nonpolar molecules interact through weak cation induced dipole interactions. Therefore the higher number of available cations dominates the adsorption process.^18^

A decrease in temperature to 0 °C leads to an increase in loading on both zeolites, which is significantly more pronounced for propane than for DME (see Figure 3). This is due to the fact that DME already reaches nearly its saturation capacity at 25 °C. In addition, the energetic values of the adsorption sites become more important at lower temperatures. The reduced kinetic energy of the molecules allows optimized orientation toward the cations, which favors the formation of more stable interactions. In the initial concentration range, the loading of DME on zeolite NaX is slightly higher than the loading on zeolite CaNaX as it was already the case at 25 °C. This may indicate once again that the higher number of Na^+^ ions could outweigh the higher energetic value of the Ca^2+^ ions. As the temperature decreases the isotherms of both materials move closer together because the divalent Ca^2+^ ions in zeolite CaNaX compensate the lower number of cations through stronger electrostatic interactions. The energetic heterogeneity of zeolite CaNaX is confirmed by the trend of the adsorption enthalpy, which decreases from 130 to 60 kJ/mol in the loading range investigated.

At −20 °C the DME loadings on both zeolites are approximately 70 mc/uc, and therefore comparable in scale. This may be because the alignment of the DME molecules can now occur in an even more targeted manner due to their low kinetic energy and the resulting, on average, longer duration of optimal alignment with the energetically more valuable Ca^2+^ ions. The higher valence of Ca^2+^ ions compared to Na^+^ ions fully compensates the lower number of cations. Under these assumptions, individual Ca^2+^ ions can bind more DME molecules at the same time, which means the molecules are more strongly fixed than at Na^+^ ions which have a single charge. Synergistically the lower temperatures enhance both the cation–dipole interactions and the lateral dipole–dipole interactions between the DME molecules. This energetically more stable configuration results in the molecules being packed more tightly and thus arranged in a more coordinated manner around the Ca^2+^ ions.

In general, DME benefits significantly more from the exchange for the energetically more valuable Ca^2+^ ions than propane. In the initial range the adsorption behavior of DME, particularly on zeolite NaX, is dominated by the number of available cations, while with increasing coverage the energetic value of the Ca^2+^ ions and the formation of additional lateral dipole–dipole interactions become decisive.

### 3.2 Binary Adsorption of Propane and DME on Zeolites NaX and CaNaX

Figure 5 shows the measured pure component data (filled symbols), the isotherm fits (solid lines), and the experimentally determined binary mixture data (open symbols) for propane and DME in the temperature range from −20 to 25 °C on the zeolites NaX (left) and CaNaX (right). In addition, the predicted mixture isotherms using the IAST model are shown as dashed lines. The experimental determination of the mixture isotherms was performed at a constant partial pressure of 2000 Pa across all equilibrium steps, with the molar fractions of the adsorptives varying over the range between zero and one. This experimental procedure delivers insights into competing adsorption between the two components at a nearly constant surface coverage.

**Figure 5:**
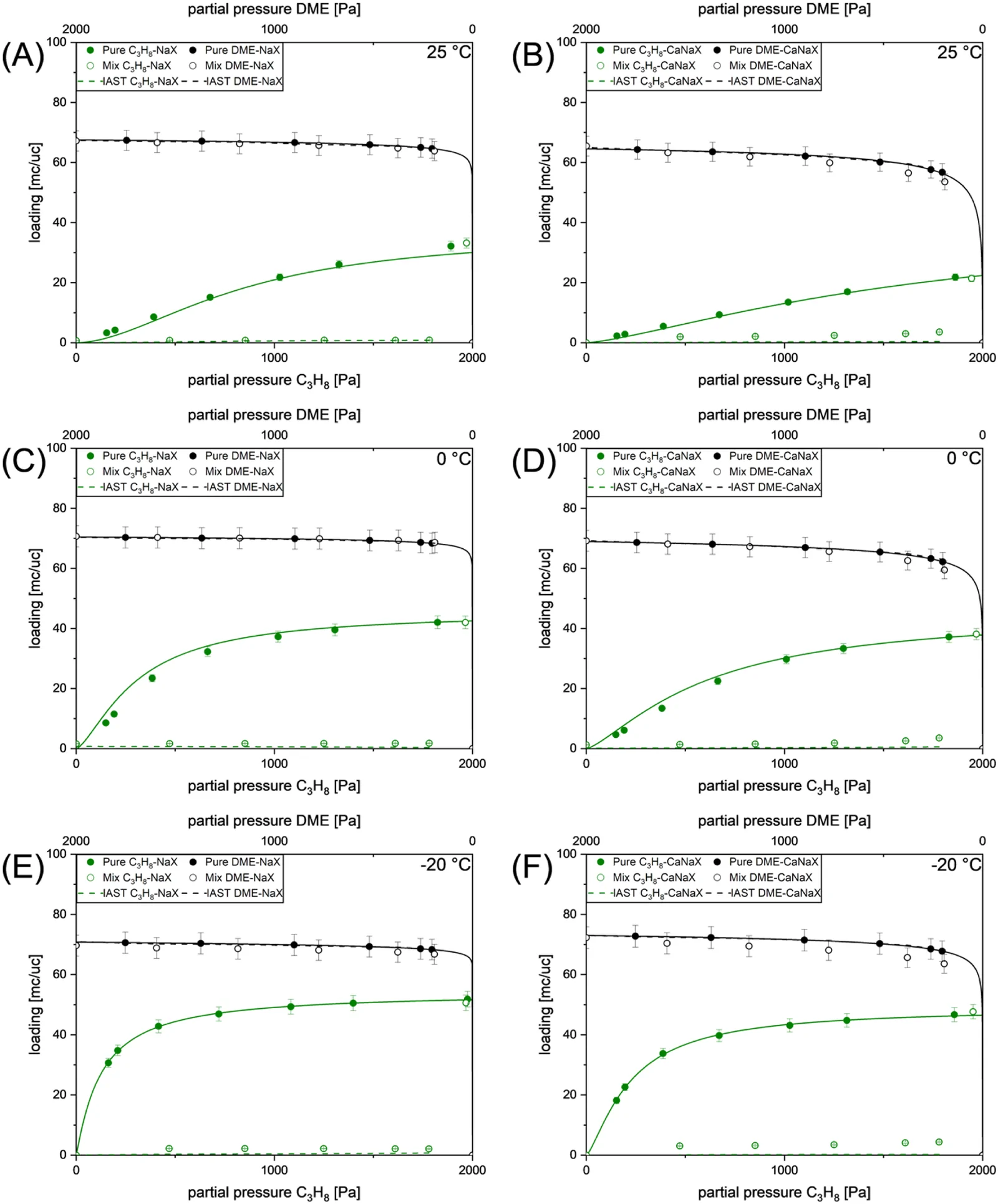
Pure component (filled symbols) and mixture adsorption isotherms (open symbols) of propane (green) and dimethyl ether (black) on zeolites NaX (A, C, E) and CaNaX (B, D, F) at 25 °C (A, B), 0 °C (C, D), and −20 °C (E, F) with constant total partial pressure of 2000 Pa per equilibrium step. Solid lines: pure component isotherm fit using the Sips equation. Dashed lines: IAST predictions.

Mixture adsorption shows significant differences compared to pure component adsorption resulting from the competition for available adsorption sites. For both zeolites, a pronounced selectivity in favor of DME is observed at all three temperatures across the entire partial pressure range. DME loading in the mixture nearly reaches the pure component loading. On the other hand, propane is almost completely displaced by the DME molecules. As already explained in section 3.1 the adsorption of DME is dominated by strong cation–dipole interactions, whereas propane can form only weaker, induced dipole interactions with the cations as well as dispersion and induced dipole interactions with the zeolite lattice. The nearly complete displacement of propane in the mixture suggests strong competition between the two adsorptives for the same adsorption sites. Consequently, the cations provide the most attractive adsorption sites for propane as well.

At a temperature of 25 °C, both zeolites exhibit nearly complete selectivity for DME (see Figure 5A and B). While DME on zeolite NaX reaches its pure component capacity with approximately 65 mc/uc almost exactly at the lowest partial pressure of 200 Pa, the propane loading reaches values below 1 mc/uc. This is due to the significantly higher affinity of the permanent DME dipole to the positively charged Na^+^ ions. This results in significantly stronger interactions, which is also confirmed by the high difference in adsorption enthalpies. The kinetic energy at 25 °C favors the flexibility of the adsorbing molecule, thereby facilitating diffusion through the narrow apertures to the active sites. Due to the high number of cations in zeolite NaX the DME molecules can form interactions with cations even at high loadings. This results in a high packing density with strong lateral interactions between the DME molecules. The propane molecules are unable to disrupt the arrangement of the DME molecules. The experimentally determined propane loading of 1 mc/uc lies within the range of the measurement error (cf. Figure 5A).

Zeolite CaNaX also exhibits very high selectivity for the polar DME molecules. The exchange of monovalent Na^+^ ions for divalent Ca^2+^ ions leads to even more attractive interactions. Despite the stronger interactions with the Ca^2+^ ions, slightly lower DME loadings are observed on zeolite CaNaX compared to the pure component isotherm. In the following, we discuss a very small effect that is nevertheless significantly larger than the measurement uncertainty. This effect occurs at low DME and thus high propane partial pressures. At a partial pressure of about 200 Pa (in the presence of 1800 Pa of propane), DME reaches loadings of approximately 60 mc/uc, compared to approximately 64 mc/uc in pure component adsorption. On the other hand, propane achieves a loading of about 5 mc/uc (cf. Figure 5B). To verify that the residual propane loading was not caused by the applied equilibrium criterion, the system was maintained under these conditions for 15 h. No measurable change in the outlet concentrations was observed during this extended period. The term metastable describes a state that remains unchanged on the experimentally accessible time scale and is not intended as proof of a thermodynamically characterized local minimum. This suggests that, even at 25 °C, in addition to the energetic affinity (three- to four-times) higher adsorption enthalpy of DME compared to propane (cf. Figure 4B), other effects influence adsorption.

At the initial measurement point, the partial pressure of propane is 1800 Pa, while that of DME is approximately 200 Pa. Consequently, there are significantly more propane molecules than DME molecules in the gas phase at the starting point. The higher initial adsorption enthalpies of propane on zeolite CaNaX compared to zeolite NaX indicate that the first propane molecules form stronger interactions with the Ca^2+^ cations compared to the Na^+^ cations in zeolite NaX and therefore approach the Ca^2+^ cations more closely. It is conceivable that in the subsequent concentration steps, this close coordination of propane molecules leads to a shielding of the Ca^2+^ cations and hinders the energetically optimal orientation of the DME molecules at the Ca^2+^ cations. Under these assumptions, the adsorbing DME molecules then trap propane molecules instead of displacing them. Unlike in zeolite NaX the dense structure of DME molecules now contains some propane molecules, and the mixture measurement therefore shows a nearly constant propane loading. In a thermodynamic sense this would not be a state of equilibrium at the energetic optimum, but would represent a metastable state due to sterically hindered displacement of propane molecules.

Lowering the temperature to 0 °C results in similar adsorption behavior as in case of 25 °C. The lower temperature even enhances the alignment of the adsorptive to the adsorption sites. At the same time, the degree of coverage increases slightly due to the increasing strength of the interactions.

On zeolite NaX, DME continues to reach nearly its pure component loading of approximately 70 mc/uc. Similarly, adsorption of propane is almost completely suppressed (cf. Figure 5C), so that the propane loading is less than 1 mc/uc.

On zeolite CaNaX there is once again a slight deviation of the DME loading in the mixture from the data of the pure components. At a low partial pressure of about 200 Pa (in the presence of 1800 Pa propane), DME reaches a loading of about 65 mc/uc, while propane shows a residual loading of about 5 mc/uc (cf. Figure 5D). The deviations from the corresponding pure component loadings are of similar magnitude compared to 25 °C, suggesting that the mechanisms discussed for 25 °C are also present at 0 °C. Since diffusion within the zeolite framework is slower at 0 °C than at 25 °C and propane comes even closer to the Ca^2+^ cations, optimal alignment and packing of the DME molecules is more difficult due to the propane molecules already adsorbed. At the same time, DME can form stronger interactions with the Ca^2+^ cations, and the lateral dipole–dipole interactions between the DME molecules become stronger. These opposing effects appear to compensate each other when the temperature is lowered from 25 to 0 °C, so that adsorption of propane is similar to that at 25 °C.

When the temperature is further lowered to −20 °C, propane continues to be almost completely displaced from zeolite NaX, and DME continues to reach the saturation loading of approximately 70 mc/uc observed in the pure component experiments (cf. Figure 5E). It can thus be concluded that the strength of the interactions with Na^+^ ions is insufficient to adsorb propane in a mixture with DME, even at low temperatures.

At the same time, on zeolite CaNaX, the effect described at 25 and 0 °C is more pronounced. At a partial pressure of approximately 200 Pa DME (in the presence of 1800 Pa propane), DME achieves a loading of only about 63 mc/uc, whereas the loading of the pure component is approximately 70 mc/uc. The propane loading in the mixture increases to about 8–10 mc/uc (cf. Figure 5F). While the opposing effects appear to compensate for one another when the temperature is lowered from 25 to 0 °C, lowering the temperature from 0 °C to −20 °C apparently enhances the steric effects.

To quantify the preferential adsorption of DME, the experimental DME/propane selectivities were evaluated at equal partial pressures of p_DME_ = *p*_Propane_ = 1000 Pa. For zeolite NaX, selectivities of approximately 81, 41, and 32 were obtained at 25 °C, 0 °C, and −20 °C, respectively. The corresponding values for zeolite CaNaX were approximately 31, 43, and 22. All values clearly demonstrate the preferential adsorption of DME, while the lower selectivity of CaNaX at −20 °C is consistent with the more pronounced residual propane loading observed under these conditions.

The experimental results are supported by theoretical modeling using IAST, as well as supplementary experiments on mixture adsorption at equimolar composition with varying coverage (see SI file, S5). Qualitatively, the IAST model accurately reflects the system behavior. In particular, the pronounced selectivity in favor of DME as well as the nearly complete displacement of propane are correctly predicted. The metastable states during adsorption on zeolite CaNaX cannot be described by a thermodynamically based theory like IAST. Other deviations attributable to the mathematical sensitivity of the IAST to the isotherm profile at very low loading levels cannot be ruled out. As DME, due to strong interactions, already reaches the saturation plateau at the first equilibrium point and no measurement points lie within the steep rise of the isotherm, the computational precision of the selectivity prediction is limited. The proposed adsorption mechanisms in the mixture are supported by the mixture adsorption experiments at equimolar composition shown in the Supporting Information (see SI file, Figure S2). Since these measurements consistently reflect the described relationships between energetic preference and steric hindrance, a detailed discussion in the main text was omitted in favor of a focused presentation of the results.

## 4 Summary and Conclusion

In this study, the influence of ether (C–O–C) and aliphatic (C–H) functionalities on competitive adsorption was investigated. Therefore, a binary mixture of propane and DME was adsorbed on FAU-type X zeolites NaX and CaNaX at temperatures between −20 and 25 °C

Pure component measurements show that DME exhibits significantly higher loadings than propane over the entire concentration range. While DME reaches almost its saturation loading at a low relative pressure (200 Pa) and exhibits a distinct saturation plateau, the loading of propane increases continuously as the relative pressure increases. Both can be attributed to the permanent dipole moment of DME, which enables strong cation–dipole interactions with the extra-framework cations of the zeolites. These interactions are particularly pronounced on zeolite CaNaX due to the higher charge density of Ca^2+^ ions. In contrast, propane interacts mainly via weaker dispersion and induced dipole interactions. While propane benefits from a higher number of accessible cations in zeolite NaX, DME profits more strongly from the higher energetic quality of Ca^2+^ sites.

The mixture experiments clearly demonstrate a strong competitive adsorption, with a pronounced selectivity toward DME on both zeolites. DME largely reaches its pure-component loading, whereas propane is almost completely displaced under most conditions. This highlights the dominant role of strong cation–dipole interactions over weaker induced interactions. On zeolite CaNaX kinetic limitations hinder the complete displacement of initially adsorbed propane molecules, resulting in residual propane loadings and a slight reduction in DME capacity. These findings indicate that, in addition to energetic effects, molecular packing and mobility within the pore system play a role which may lead to a metastable state.

The IAST model was able to qualitatively predict the adsorption behavior and the strong selectivity toward DME. However, systematic deviations occur in adsorption on zeolite CaNaX at low DME loadings, where equilibrium is disturbed by kinetic limitations.

## Supplementary Material



## Notes

Concentration profiles for binary mixture experiments; derivation of the formulas to calculate the pure component and mixture loadings; table of the uncertainties of the apparatus used and detailed calculation of the statistical error following Gaussian error propagation; adsorption data of the experiments; determination of load-dependent heats of adsorption; XRPD/Rietveld refinement results for NaX and CaNaX (PDF)
