# Supplementary material for: The Influence of C–O–C and C–H Functionalities on Competitive Adsorption of Dimethyl Ether and Propane on FAU-Type X Zeolites
Source: ACS Omega. 2026 Sep 4;11(37):56336–45. doi: 10.1021/acsomega.6c06435 (PMC13613419; doi:10.1021/acsomega.6c06435)
Supplement: Supplementary file 1 [file ao6c06435_si_001.pdf]

Supporting Information

The influence of C-O-C and C-H functionalities on competitive adsorption of dimethyl ether and propane on FAU-type X zeolites

Malte Menk[*,a], *Christian Bläker*[a], *Christoph Pasel*[a], *Dieter Bathen*[a,b]

[a] Thermal Process Engineering, University of Duisburg-Essen, Lotharstraße 1, D-47057 Duisburg, Germany

[b] Institute for Environment & Energy, Technology & Analytics e. V. (IUTA), Bliersheimer Straße 58-60, D-47229 Duisburg, Germany

*E-mail: malte.menk@uni-due.de

S1. Concentration profiles for binary mixture experiments

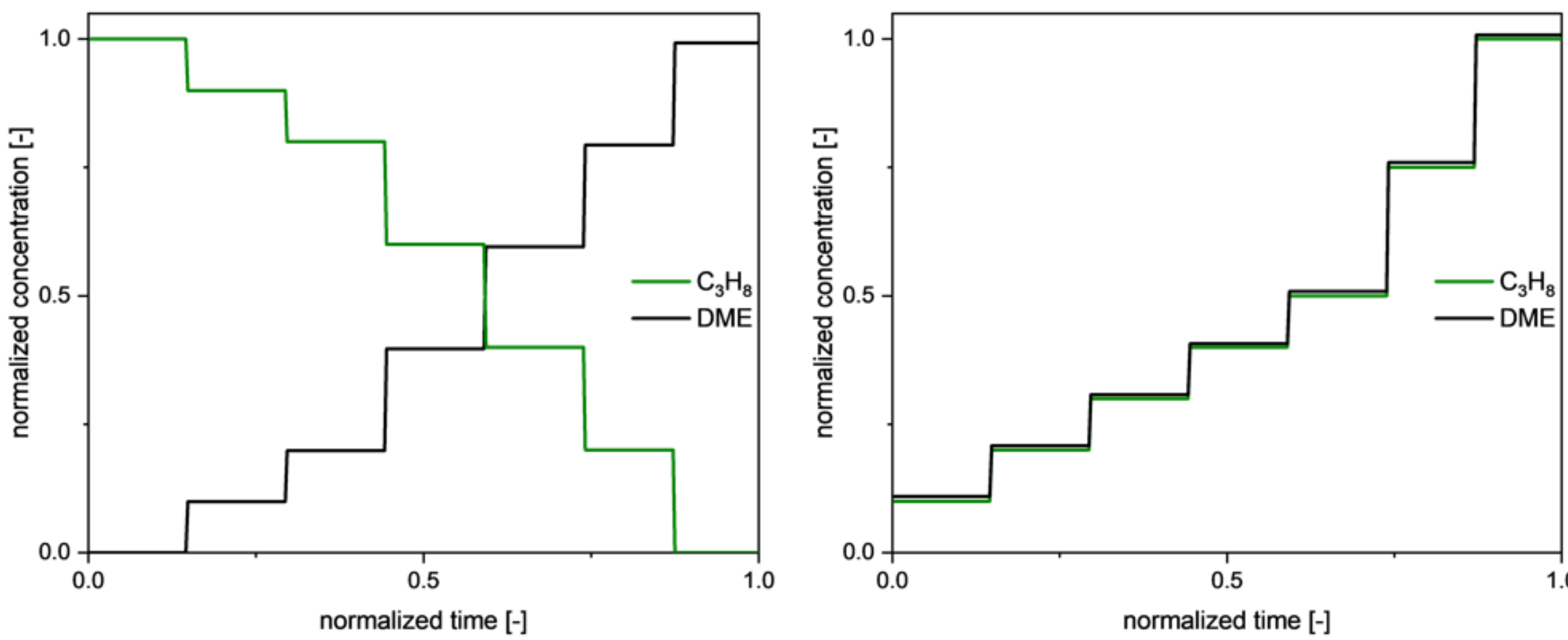

Figure S1: Exemplary concentration profile for a binary mixture experiment with constant total concentration (left) per equilibrium step and equimolar concentration increments (right) per equilibrium step.

S2. Derivation of the formula to calculate the pure component loading

Mass balance around the adsorber:

$$\dot{n}_{M,in} + \dot{n}_{1,in} = \dot{n}_{M,out} + \dot{n}_{1,out} + \dot{n}_{1,ads} \tag{S1}$$

Assumption: No adsorption of matrix gas

$$\dot{n}_{M,in} = \dot{n}_{M,out} \tag{S2}$$

$$\dot{n}_{1,in} = \dot{n}_{1,out} + \dot{n}_{1,ads} \tag{S3}$$

Inlet and outlet streams are determined by the product of total streams and the mole fraction of the adsorptive:

$$\dot{n}_{1,in} = \dot{n}_{tot,in} \cdot y_{1,in} \tag{S4}$$

$$\dot{n}_{1,out} = \dot{n}_{tot,out} \cdot y_{1,out} \tag{S5}$$

(S4) and (S5) in (S3)

$$\dot{n}_{1,ads} = \dot{n}_{tot,in} \cdot y_{1,in} - \dot{n}_{tot,out} \cdot y_{1,out} \tag{S6}$$

Total mass balance:

$$\dot{n}_{tot,out} = \dot{n}_{tot,in} - \dot{n}_{1,ads} \tag{S7}$$

(S7) in (S6):

$$\dot{n}_{1,ads} = \dot{n}_{tot,in} \cdot \frac{y_{1,in} - y_{1,out}}{1 - y_{1,out}} \tag{S8}$$

Integration of (S8):

$$n_{1,ads} = \dot{n}_{tot,in} \int_0^t \frac{y_{1,in} - y_{1,out}}{1 - y_{1,out}} dt \tag{S9}$$

Calculation of the loading:

$$X_{eq} = \frac{n_{1,ads}}{m_{adsorbent}} = \frac{\dot{n}_{tot,in}}{m_{adsorbent}} \int_0^t \frac{y_{1,in} - y_{1,out}}{1 - y_{1,out}} dt \tag{S10}$$

S3. Derivation of the formula to calculate the loading of component 1 in a binary mixture measurement

Mass balance around the adsorber:

$$\dot{n}_{M,in} + \dot{n}_{1,in} + \dot{n}_{2,in} = \dot{n}_{M,out} + \dot{n}_{1,out} + \dot{n}_{2,out} + \dot{n}_{1,ads} + \dot{n}_{2,ads} \quad \text{(S11)}$$

Assumption: No adsorption of matrix gas

$$\dot{n}_{M,in} = \dot{n}_{M,out} \quad \text{(S12)}$$

$$\dot{n}_{1,in} + \dot{n}_{2,in} = \dot{n}_{1,out} + \dot{n}_{2,out} + \dot{n}_{1,ads} + \dot{n}_{2,ads} \quad \text{(S13)}$$

Total in- and outlet streams:

$$\dot{n}_{tot,in} = \dot{n}_{1,in} + \dot{n}_{2,in} \quad \text{(S14)}$$

$$\dot{n}_{tot,out} = \dot{n}_{1,out} + \dot{n}_{2,out} \quad \text{(S15)}$$

Inlet and outlet streams are determined by the product of total streams and the mole fraction of the adsorptives:

$$\dot{n}_{1,in} = \dot{n}_{tot,in} \cdot y_{1,in} \quad \text{(S16)}$$

$$\dot{n}_{2,in} = \dot{n}_{tot,in} \cdot y_{2,in} \quad \text{(S17)}$$

$$\dot{n}_{1,out} = \dot{n}_{tot,out} \cdot y_{1,out} \quad \text{(S18)}$$

$$\dot{n}_{2,out} = \dot{n}_{tot,out} \cdot y_{2,out} \quad \text{(S19)}$$

Mass balances of each component and total mass balance:

$$\dot{n}_{1,ads} = \dot{n}_{1,in} - \dot{n}_{1,out} = \dot{n}_{tot,in} \cdot y_{1,in} - \dot{n}_{tot,out} \cdot y_{1,out} \quad \text{(S20)}$$

$$\dot{n}_{2,ads} = \dot{n}_{2,in} - \dot{n}_{2,out} = \dot{n}_{tot,in} \cdot y_{2,in} - \dot{n}_{tot,out} \cdot y_{2,out} \quad \text{(S21)}$$

$$\dot{n}_{tot,out} = \dot{n}_{tot,in} - \dot{n}_{1,ads} - \dot{n}_{2,ads} \quad \text{(S22)}$$

(S20) and (S21) in (S22):

$$\dot{n}_{tot,out} = \dot{n}_{tot,in} \cdot \frac{1 - y_{1,in} - y_{2,in}}{1 - y_{1,out} - y_{2,out}} \quad \text{(S23)}$$

(S23) in (S20):

$$\dot{n}_{1,ads} = \dot{n}_{tot,in} \cdot \left(y_{1,in} - y_{1,out} \frac{1 - y_{1,in} - y_{2,in}}{1 - y_{1,out} - y_{2,out}} \right) \tag{S24}$$

Integration of (S24):

$$\dot{n}_{1,ads} = \dot{n}_{tot,in} \cdot \int_0^t \left(y_{1,in} - y_{1,out} \frac{1 - y_{1,in} - y_{2,in}}{1 - y_{1,out} - y_{2,out}} \right) dt \tag{S25}$$

Calculation of the loading:

$$X_{eq} = \frac{n_{1,ads}}{m_{adsorbent}} = \frac{\dot{n}_{tot,in}}{m_{adsorbent}} \int_0^t \left(y_{1,in} - y_{1,out} \frac{1 - y_{1,in} - y_{2,in}}{1 - y_{1,out} - y_{2,out}} \right) dt \tag{S26}$$

S4. Experimental uncertainties and error propagation

Table S1: Statistical uncertainties of the apparatuses used.

Apparatus	Measured variable	Statistical Uncertainty
FT-IR	Concentration	± 1.5 %
MFC	Mass flow rate	± 0.20 %
Balance	Adsorbent mass	± 0.03 mg

Statistical error of amount of component 1 adsorbed following the Gaussian error propagation:

$$\sigma_y = \sqrt{\sum_i \left[\left(\frac{\partial y}{\partial x_i} \right)^2 \cdot \sigma_{x_i}^2 \right]} \tag{S27}$$

$$X_{eq,1} = \frac{n_1}{m_{Ad}} = \frac{\dot{n}_{tot,in}}{m_{Ad}} \cdot \sum_{j=1}^{j_{eq}} \left[\left(y_{1,in} - \frac{y_{1,out,j} \cdot (1 - y_{1,in} - y_{2,in})}{1 - y_{1,out,j} - y_{2,out,j}} \right) \cdot \Delta t_j \right] \tag{S28}$$

$$\frac{\partial X_{eq,1}}{\partial m_{Ad}} = \frac{-1}{m_{Ad}^2} \cdot \sum_{j=1}^{j_{eq}} \left[\left(y_{1,in} - \frac{y_{1,out,j} \cdot (1 - y_{1,in} - y_{2,in})}{1 - y_{1,out,j} - y_{2,out,j}} \right) \cdot \Delta t_j \right] \tag{S29}$$

$$\frac{\partial X_{eq,1}}{\partial \dot{n}_{tot,in}} = \frac{1}{m_{Ad}} \cdot \sum_{j=1}^{j_{eq}} \left[\left(y_{1,in} - \frac{y_{1,out,j} \cdot (1 - y_{1,in} - y_{2,in})}{1 - y_{1,out,j} - y_{2,out,j}} \right) \cdot \Delta t_j \right] \tag{S30}$$

$$\frac{\partial X_{eq,1}}{\partial y_{1,in}} = \frac{\dot{n}_{tot,in}}{m_{Ad}} \cdot \sum_{j=1}^{j_{eq}} \left[\left(1 - \frac{y_{1,out,j}}{1 - y_{1,out,j} - y_{2,out,j}} \right) \cdot \Delta t_j \right] \tag{S31}$$

$$\frac{\partial X_{eq,1}}{\partial y_{2,in}} = \frac{\dot{n}_{tot,in}}{m_{Ad}} \cdot \sum_{j=1}^{j_{eq}} \left[\left(\frac{- y_{1,out,j}}{1 - y_{1,out,j} - y_{2,out,j}} \right) \cdot \Delta t_j \right] \tag{S32}$$

$$\frac{\partial X_{eq,1}}{\partial y_{1,out}} = \frac{\partial X_{eq,1}}{\partial y_{2,out}} = \frac{\dot{n}_{tot,in}}{m_{Ad}} \cdot \sum_{j=1}^{j_{eq}} \left[\left(- \frac{(1 - y_{1,in} - y_{2,in}) \cdot (1 - y_{2,out,j})}{(1 - y_{1,out,j} - y_{2,out,j})^2} \right) \cdot \Delta t_j \right] \tag{S33}$$

S5. Adsorption isotherm data

Table S2: Pure component isotherm data of propane on zeolite NaX at -20 °C.

p [Pa]	X [mc/uc]	X [mol/kg]
152.32	30.68	1.75
195.38	34.8	1.99
388.96	42.85	2.45
670.64	46.92	2.68
1025.99	49.33	2.82
1314.23	50.51	2.89
1855.10	51.82	2.96

Table S3: Pure component isotherm data of propane on zeolite CaNaX at -20 °C.

p [Pa]	X [mc/uc]	X [mol/kg]
152.72	18.16	1.04
195.47	22.61	1.29
387.01	33.78	1.93
670.21	39.74	2.27
1026.73	43.14	2.47
1314.53	44.78	2.56
1856.69	46.73	2.67

Table S4: Pure component isotherm data of propane on zeolite NaX at 0 °C.

p [Pa]	X [mc/uc]	X [mol/kg]
149.64	8.58	0.49
191.6	11.51	0.66
383.81	23.46	1.34
663.29	32.3	1.85
1019.06	37.27	2.13
1305.47	39.55	2.26
1823.86	42.06	2.40

Table S5: Pure component isotherm data of propane on zeolite CaNaX at 0 °C.

p [Pa]	X [mc/uc]	X [mol/kg]
149.28	4.67	0.27
191.22	6.13	0.35
381.89	13.43	0.77
663.16	22.47	1.28
1009.67	29.73	1.70
1297.33	33.34	1.91
1829.63	37.21	2.13

Table S6: Pure component isotherm data of propane on zeolite NaX at 25 °C.

p [Pa]	X [mc/uc]	X [mol/kg]
152.66	2.88	0.16
193.43	3.52	0.20
384.71	7.18	0.41
667.03	13.02	0.74
1027.32	19.73	1.13
1315.69	23.87	1.36
1859.38	29.41	1.68

Table S7: Pure component isotherm data of propane on zeolite CaNaX at 25 °C.

p [Pa]	X [mc/uc]	X [mol/kg]
152.41	2.31	0.13
195.33	2.91	0.17
389.00	5.53	0.32
667.07	9.23	0.53
1027.59	14.08	0.80
1317.22	17.35	0.99
1860.29	22.26	1.27

Table S8: Pure component isotherm data of DME on zeolite NaX at -20 °C.

p [Pa]	X [mc/uc]	X [mol/kg]
204.20	68.30	3.90
261.44	68.57	3.92
518.47	69.30	3.96
896.81	69.87	3.99
1366.25	70.36	4.02
1751.09	70.61	4.03
2510.01	71.08	4.06

Table S9: Pure component isotherm data of DME on zeolite CaNaX at -20 °C.

p [Pa]	X [mc/uc]	X [mol/kg]
204.79	67.78	3.87
261.95	68.51	3.92
519.44	70.27	4.02
900.08	71.46	4.08
1370.79	72.32	4.13
1752.39	72.76	4.16
2508.70	73.41	4.20

Table S10: Pure component isotherm data of DME on zeolite NaX at 0 °C.

p [Pa]	X [mc/uc]	X [mol/kg]
204.20	68.34	3.91
261.44	68.64	3.92
518.47	69.33	3.96
896.81	69.91	3.99
1366.25	70.08	4.00
1751.09	70.30	4.02
2510.01	70.70	4.04

Table S11: Pure component isotherm data of DME on zeolite CaNaX at 0 °C.

p [Pa]	X [mc/uc]	X [mol/kg]
202.06	62.21	3.55
261.19	63.27	3.62
517.81	65.45	3.74
895.49	66.95	3.83
1363.64	68.05	3.89
1745.35	68.63	3.92
2499.80	69.46	3.97

Table S12: Pure component isotherm data of DME on zeolite NaX at 25 °C.

p [Pa]	X [mc/uc]	X [mol/kg]
204.37	64.62	3.69
261.37	65.01	3.72
518.20	65.93	3.77
897.29	66.64	3.81
1365.48	67.13	3.84
1745.39	67.40	3.85
2503.11	67.79	3.87

Table S13: Pure component isotherm data of DME on zeolite CaNaX at 25 °C.

p [Pa]	X [mc/uc]	X [mol/kg]
204.55	56.75	3.24
261.06	57.64	3.29
517.97	60.14	3.44
895.09	62.12	3.55
1363.09	63.57	3.63
1742.52	64.32	3.68
2498.87	65.48	3.74

Table S14: Mixture isotherm data of propane and DME with equimolar concentration increments per equilibrium step on zeolite NaX at -20 °C.

p_{C3H8} [Pa]	X_{C3H8} [mc/uc]	X_{C3H8} [mol/kg]	$IAST_{C3H8}$ [mc/uc]	$IAST_{C3H8}$ [mol/kg]	p_{DME} [Pa]	X_{DME} [mc/uc]	X_{DME} [mol/kg]	$IAST_{DME}$ [mc/uc]	$IAST_{DME}$ [mol/kg]
162.92	2.61	0.15	0.06	0.00	202.79	69.00	3.94	67.94	3.88
207.31	2.52	0.14	0.05	0.00	259.09	69.28	3.96	68.39	3.91
413.77	2.42	0.14	0.05	0.00	512.75	69.98	4.00	69.41	3.97
717.51	2.31	0.13	0.04	0.00	884.14	70.46	4.03	70.02	4.00
1090.62	2.22	0.13	0.03	0.00	1339.60	70.87	4.05	70.38	4.02
1390.06	2.11	0.12	0.03	0.00	1706.21	71.05	4.06	70.56	4.03
1984.37	2.11	0.12	0.03	0.00	2431.56	71.38	4.08	70.79	4.05

Table S15: Mixture isotherm data of propane and DME with equimolar concentration increments per equilibrium step on zeolite CaNaX at -20 °C.

p_{C3H8} [Pa]	X_{C3H8} [mc/uc]	X_{C3H8} [mol/kg]	$IAST_{C3H8}$ [mc/uc]	$IAST_{C3H8}$ [mol/kg]	p_{DME} [Pa]	X_{DME} [mc/uc]	X_{DME} [mol/kg]	$IAST_{DME}$ [mc/uc]	$IAST_{DME}$ [mol/kg]
162.82	4.57	0.26	0.00	0.00	202.36	68.08	3.89	67.75	3.87
208.99	4.69	0.27	0.00	0.00	258.42	68.83	3.93	68.44	3.91
413.66	4.69	0.27	0.00	0.00	510.81	70.67	4.04	70.19	4.01
715.91	4.69	0.27	0.00	0.00	882.79	71.90	4.11	71.42	4.08
1086.36	4.58	0.26	0.00	0.00	1339.04	72.76	4.16	72.26	4.13
1386.72	4.49	0.26	0.00	0.00	1704.86	73.18	4.18	72.71	4.16
1977.39	4.49	0.26	0.00	0.00	2428.20	73.83	4.22	73.33	4.19

Table S16: Mixture isotherm data of propane and DME with equimolar concentration increments per equilibrium step on zeolite NaX at 0 °C.

p_{C3H8} [Pa]	X_{C3H8} [mc/uc]	X_{C3H8} [mol/kg]	$IAST_{C3H8}$ [mc/uc]	$IAST_{C3H8}$ [mol/kg]	p_{DME} [Pa]	X_{DME} [mc/uc]	X_{DME} [mol/kg]	$IAST_{DME}$ [mc/uc]	$IAST_{DME}$ [mol/kg]
162.44	1.81	0.10	0.01	0.00	202.45	69.07	3.95	67.94	3.88
208.12	1.79	0.10	0.01	0.00	258.77	69.37	3.96	68.39	3.91
416.34	1.76	0.10	0.01	0.00	512.12	70.06	4.00	69.41	3.97
718.12	1.66	0.09	0.00	0.00	883.26	70.60	4.03	70.02	4.00
1091.01	1.55	0.09	0.00	0.00	1339.44	71.05	4.06	70.38	4.02
1391.07	1.47	0.08	0.00	0.00	1706.21	71.26	4.07	70.56	4.03
1986.80	1.30	0.07	0.00	0.00	2434.17	71.58	4.09	70.79	4.05

Table S17: Mixture isotherm data of propane and DME with equimolar concentration increments per equilibrium step on zeolite CaNaX at 0 °C.

p_{C3H8} [Pa]	X_{C3H8} [mc/uc]	X_{C3H8} [mol/kg]	$IAST_{C3H8}$ [mc/uc]	$IAST_{C3H8}$ [mol/kg]	p_{DME} [Pa]	X_{DME} [mc/uc]	X_{DME} [mol/kg]	$IAST_{DME}$ [mc/uc]	$IAST_{DME}$ [mol/kg]
161.75	2.50	0.14	0.00	0.00	200.08	60.88	3.48	62.73	3.58
207.72	2.62	0.15	0.00	0.00	257.50	61.75	3.53	63.57	3.63
413.67	2.69	0.15	0.00	0.00	509.96	63.87	3.65	65.71	3.76
716.19	2.69	0.15	0.00	0.00	880.37	65.41	3.74	67.24	3.84
1088.63	2.59	0.15	0.00	0.00	1335.81	66.45	3.80	68.29	3.90
1388.95	2.50	0.14	0.00	0.00	1704.46	66.97	3.83	68.86	3.93
1981.13	2.36	0.13	0.00	0.00	2426.60	67.77	3.87	69.63	3.98

Table S18: Mixture isotherm data of propane and DME with equimolar concentration increments per equilibrium step on zeolite NaX at 25 °C.

p_{C3H8} [Pa]	X_{C3H8} [mc/uc]	X_{C3H8} [mol/kg]	$IAST_{C3H8}$ [mc/uc]	$IAST_{C3H8}$ [mol/kg]	p_{DME} [Pa]	X_{DME} [mc/uc]	X_{DME} [mol/kg]	$IAST_{DME}$ [mc/uc]	$IAST_{DME}$ [mol/kg]
161.60	0.90	0.05	0.24	0.01	202.46	64.21	3.67	63.80	3.65
207.85	0.92	0.05	0.20	0.01	257.64	64.64	3.69	64.83	3.70
415.40	0.92	0.05	0.12	0.01	510.01	65.55	3.75	66.46	3.80
718.93	0.88	0.05	0.07	0.00	880.31	66.23	3.78	67.02	3.83
1091.83	0.79	0.05	0.05	0.00	1335.86	66.76	3.81	67.24	3.84
1391.79	0.70	0.04	0.04	0.00	1700.75	67.00	3.83	67.33	3.85
1986.71	0.70	0.04	0.03	0.00	2424.36	67.40	3.85	67.41	3.85

Table S19: Mixture isotherm data of propane and DME with equimolar concentration increments per equilibrium step on zeolite CaNaX at 25 °C.

p_{C3H8} [Pa]	X_{C3H8} [mc/uc]	X_{C3H8} [mol/kg]	$IAST_{C3H8}$ [mc/uc]	$IAST_{C3H8}$ [mol/kg]	p_{DME} [Pa]	X_{DME} [mc/uc]	X_{DME} [mol/kg]	$IAST_{DME}$ [mc/uc]	$IAST_{DME}$ [mol/kg]
162.59	2.56	0.15	0.00	0.00	202.11	54.55	3.12	56.58	3.23
207.88	2.65	0.15	0.00	0.00	258.14	55.47	3.17	57.57	3.29
413.84	2.76	0.16	0.00	0.00	510.26	58.01	3.31	60.13	3.44
715.93	2.82	0.16	0.00	0.00	879.23	60.04	3.43	61.98	3.54
1088.03	2.75	0.16	0.00	0.00	1332.89	61.48	3.51	63.27	3.62
1387.01	2.68	0.15	0.00	0.00	1698.77	62.24	3.56	63.98	3.66
1979.58	2.68	0.15	0.00	0.00	2420.54	63.31	3.62	64.95	3.71

Table S20: Mixture isotherm data of propane and DME with constant total concentration on zeolite NaX at -20 °C.

p_{C3H8} [Pa]	X_{C3H8} [mc/uc]	X_{C3H8} [mol/kg]	$IAST_{C3H8}$ [mc/uc]	$IAST_{C3H8}$ [mol/kg]	p_{DME} [Pa]	X_{DME} [mc/uc]	X_{DME} [mol/kg]	$IAST_{DME}$ [mc/uc]	$IAST_{DME}$ [mol/kg]
1966.91	50.61	2.89	50.61	2.89	0.00	0.00	0.00	0.00	0.00
1779.80	2.09	0.12	0.07	0.00	192.22	66.77	3.82	67.85	3.88
1609.59	2.12	0.12	0.03	0.00	377.27	67.45	3.85	69.04	3.94
1248.65	2.17	0.12	0.01	0.00	776.27	68.13	3.89	69.93	4.00
851.07	2.20	0.13	0.00	0.00	1183.95	68.60	3.92	70.32	4.02
469.18	2.19	0.13	0.00	0.00	1593.98	68.85	3.93	70.56	4.03
0.00	0.00	0.00	0.00	0.00	1998.50	69.67	3.98	70.71	4.04

Table S21: Mixture isotherm data of propane and DME with constant total concentration on zeolite CaNaX at -20 °C.

p_{C3H8} [Pa]	X_{C3H8} [mc/uc]	X_{C3H8} [mol/kg]	$IAST_{C3H8}$ [mc/uc]	$IAST_{C3H8}$ [mol/kg]	p_{DME} [Pa]	X_{DME} [mc/uc]	X_{DME} [mol/kg]	$IAST_{DME}$ [mc/uc]	$IAST_{DME}$ [mol/kg]
1951.43	47.66	2.72	47.66	2.72	0.00	0.00	0.00	0.00	0.00
1779.66	4.37	0.25	0.01	0.00	194.17	63.59	3.63	67.61	3.86
1609.25	4.08	0.23	0.00	0.00	379.83	65.65	3.75	69.46	3.97
1249.86	3.46	0.20	0.00	0.00	776.89	68.12	3.89	71.15	4.07
851.80	3.19	0.18	0.00	0.00	1178.67	69.47	3.97	72.01	4.12
471.34	3.07	0.18	0.00	0.00	1593.06	70.40	4.02	72.59	4.15
0.00	0.00	0.00	0.00	0.00	1999.21	72.30	4.13	73.00	4.17

Table S22: Mixture isotherm data of propane and DME with constant total concentration on zeolite NaX at 0 °C.

p_{C3H8} [Pa]	X_{C3H8} [mc/uc]	X_{C3H8} [mol/kg]	$IAST_{C3H8}$ [mc/uc]	$IAST_{C3H8}$ [mol/kg]	p_{DME} [Pa]	X_{DME} [mc/uc]	X_{DME} [mol/kg]	$IAST_{DME}$ [mc/uc]	$IAST_{DME}$ [mol/kg]
1963.82	42.00	2.40	42.00	2.40	0.00	0.00	0.00	0.00	0.00
1781.45	1.79	0.10	0.11	0.01	190.49	68.64	3.92	68.08	3.89
1610.04	1.76	0.10	0.04	0.00	374.13	69.34	3.96	69.01	3.94
1250.11	1.74	0.10	0.01	0.00	772.33	69.91	3.99	69.50	3.97
851.73	1.71	0.10	0.00	0.00	1175.35	70.09	4.01	69.66	3.98
471.67	1.68	0.10	0.00	0.00	1589.21	70.31	4.02	69.74	3.99
0.00	1.61	0.09	0.00	0.00	1997.20	70.70	4.04	69.79	3.99

Table S23: Mixture isotherm data of propane and DME with constant total concentration on zeolite CaNaX at 0 °C.

p_{C3H8} [Pa]	X_{C3H8} [mc/uc]	X_{C3H8} [mol/kg]	$IAST_{C3H8}$ [mc/uc]	$IAST_{C3H8}$ [mol/kg]	p_{DME} [Pa]	X_{DME} [mc/uc]	X_{DME} [mol/kg]	$IAST_{DME}$ [mc/uc]	$IAST_{DME}$ [mol/kg]
1968.13	38.12	2.18	38.12	2.18	0.00	0.00	0.00	0.00	0.00
1780.41	3.56	0.20	0.11	0.01	193.17	59.47	3.40	62.58	3.58
1611.40	2.60	0.15	0.04	0.00	377.96	62.59	3.58	64.81	3.70
1251.87	1.86	0.11	0.01	0.00	774.77	65.61	3.75	66.90	3.82
852.25	1.54	0.09	0.00	0.00	1175.09	67.25	3.84	67.98	3.88
471.98	1.40	0.08	0.00	0.00	1589.16	68.10	3.89	68.70	3.93
0.00	1.25	0.07	0.00	0.00	2000.62	69.20	3.95	69.21	3.96

Table S24: Mixture isotherm data of propane and DME with constant total concentration on zeolite NaX at 25 °C.

p_{C3H8} [Pa]	X_{C3H8} [mc/uc]	X_{C3H8} [mol/kg]	$IAST_{C3H8}$ [mc/uc]	$IAST_{C3H8}$ [mol/kg]	p_{DME} [Pa]	X_{DME} [mc/uc]	X_{DME} [mol/kg]	$IAST_{DME}$ [mc/uc]	$IAST_{DME}$ [mol/kg]
1970.13	33.22	1.90	33.07	1.89	0.00	0.00	0.00	0.00	0.00
1782.23	0.86	0.05	0.28	0.02	192.39	63.85	3.65	63.51	3.63
1610.80	0.83	0.05	0.08	0.00	376.96	64.81	3.70	66.05	3.77
1251.68	0.83	0.05	0.02	0.00	775.31	65.69	3.75	67.04	3.83
853.23	0.81	0.05	0.01	0.00	1175.61	66.23	3.78	67.28	3.84
471.88	0.79	0.05	0.00	0.00	1591.10	66.63	3.81	67.38	3.85
0.00	0.70	0.04	0.00	0.00	2000.00	67.23	3.84	67.44	3.85

Table S25: Mixture isotherm data of propane and DME with constant total concentration on zeolite CaNaX at 25 °C.

p_{C3H8} [Pa]	X_{C3H8} [mc/uc]	X_{C3H8} [mol/kg]	$IAST_{C3H8}$ [mc/uc]	$IAST_{C3H8}$ [mol/kg]	p_{DME} [Pa]	X_{DME} [mc/uc]	X_{DME} [mol/kg]	$IAST_{DME}$ [mc/uc]	$IAST_{DME}$ [mol/kg]
1943.84	21.41	1.22	21.41	1.22	0.00	0.00	0.00	0.00	0.00
1781.68	3.59	0.21	0.01	0.00	191.23	53.58	3.06	56.40	3.22
1611.29	3.01	0.17	0.00	0.00	374.65	56.50	3.23	59.04	3.37
1251.10	2.44	0.14	0.00	0.00	771.94	59.90	3.42	61.56	3.52
853.00	2.12	0.12	0.00	0.00	1174.48	61.90	3.54	62.89	3.59
473.61	1.99	0.11	0.00	0.00	1588.45	63.25	3.61	63.78	3.64
0.00	0.00	0.00	0.00	0.00	1999.00	65.48	3.74	64.42	3.68

Table S26: Measured load-dependent enthalpies of adsorption of C_3H_8 on NaX at 25 °C.

X_{C3H8} [mc/uc]	X_{C3H8} [mol/kg]	$\Delta h_{Ads,C3H8}$ [kJ/mol]
3.52	0.21	24.19
7.02	0.40	24.57
10.5	0.61	24.96
13.96	0.82	25.60
17.39	0.99	26.93
20.79	1.19	26.62
24.12	1.38	28.12
27.34	1.56	29.12
30.34	1.73	30.01
33.8	1.93	31.16
36.48	2.08	33.17
38.27	2.19	33.53
40.17	2.31	35.02
40.72	2.33	32.54
41.13	2.35	31.14
41.46	2.37	33.47
41.73	2.38	32.74
41.96	2.40	32.52
42.15	2.41	33.12

Table S27: Measured load-dependent enthalpies of adsorption of C_3H_8 on CaNaX at 25 °C.

X_{C3H8} [mc/uc]	X_{C3H8} [mol/kg]	$\Delta h_{Ads,C3H8}$ [kJ/mol]
3.52	0.20	32.71
6.68	0.38	31.73
10.11	0.58	30.12
13.44	0.77	28.38
16.66	0.95	26.84
19.76	1.13	26.44
22.67	1.30	25.92
25.42	1.45	27.66
28.62	1.64	28.28
31.07	1.78	29.84
32.76	1.87	31.01
34.77	1.99	31.20
35.41	2.02	32.14
35.91	2.05	31.68
36.66	2.10	32.29
37.20	2.13	32.63
37.42	2.14	30.02
37.89	2.17	29.13

Table S28: Measured load-dependent enthalpies of adsorption of DME on NaX at 25 °C.

X_{DME} [mc/uc]	X_{DME} [mol/kg]	$\Delta h_{Ads,DME}$ [kJ/mol]
2.48	0.14	95.01
4.9	0.28	91.99
7.28	0.42	89.34
11.86	0.68	87.91
16.24	0.93	89.49
18.61	1.06	88.9
23.09	1.32	89.27
27.34	1.56	92.45
31.3	1.79	93.67
35.03	2.01	93.21
39.03	2.23	96.25
42.77	2.44	94.99
46.32	2.65	95.74
49.7	2.84	95.58
52.93	3.02	96.79
56.07	3.2	97.06
59.09	3.38	95.24
62.64	3.58	96.67
66.11	3.78	97.03
69.1	3.95	94.96
70.09	4.02	87.83
70.42	4.02	69.48

Table S29: Measured load-dependent enthalpies of adsorption of DME on CaNaX at 25 °C.

X_{DME} [mc/uc]	X_{DME} [mol/kg]	$\Delta h_{Ads,DME}$ [kJ/mol]
2.49	0.14	132.32
4.92	0.28	117.5
7.32	0.42	107.9
11.95	0.68	101.81
16.38	0.94	98.83
18.77	1.07	91.58
23.32	1.33	93.57
27.63	1.58	95.79
31.7	1.81	96.98
36.17	2.07	92.36
40.25	2.3	92.41
44.01	2.51	88.7
47.48	2.71	89.28
50.78	2.9	89.1
53.88	3.08	88.62
56.83	3.25	77.1
60.3	3.45	66.06
63.27	3.62	57.99

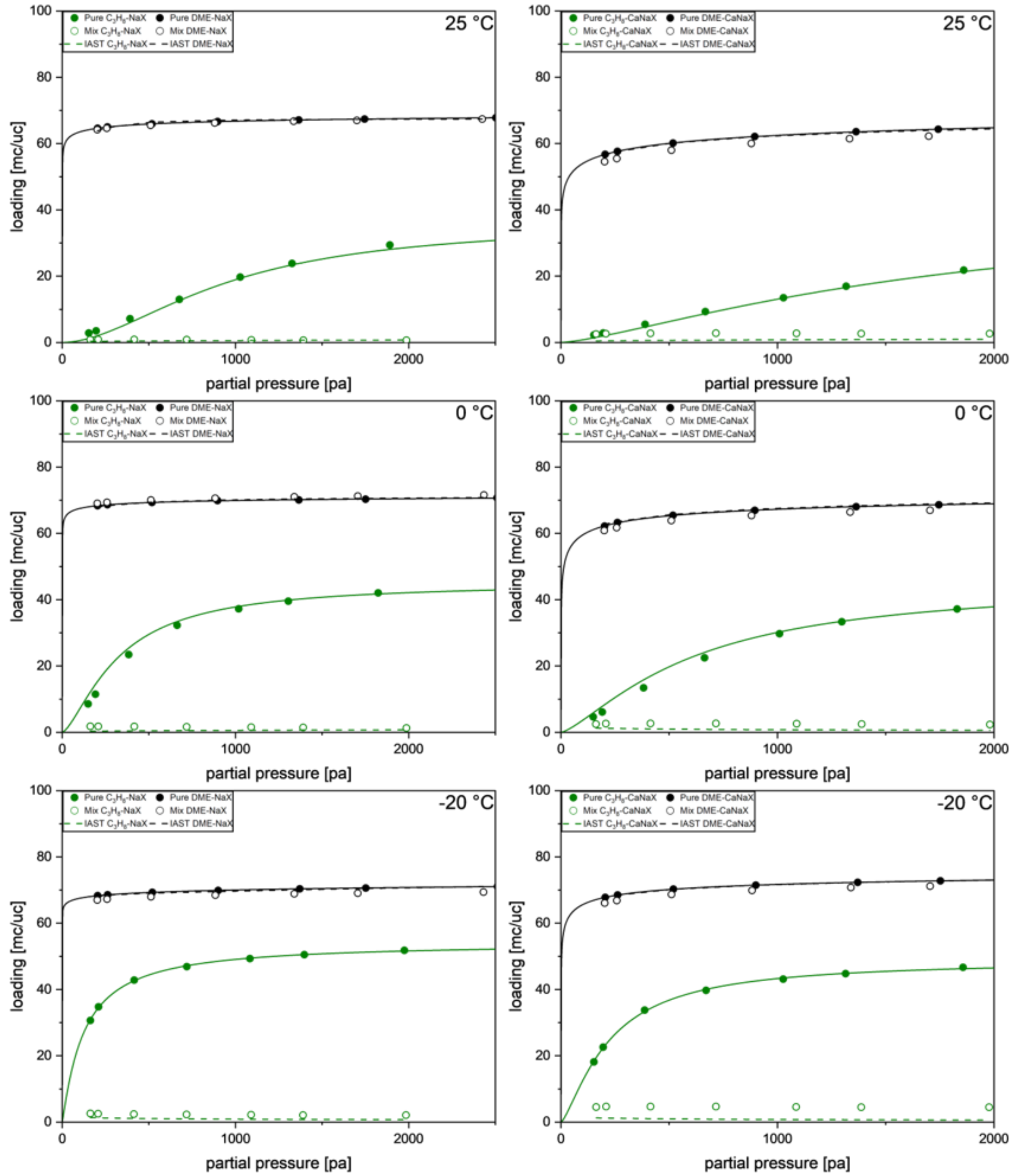

Figure S2: Pure-component (filled symbols) and mixture adsorption isotherms (open symbols) of propane (green) and DME (black) on zeolites NaX (left) at 25 °C, 0 °C and -20 °C as well as on CaNaX (right) at 25 °C, 0 °C , and -20 °C with equimolar relative pressure increments per equilibrium step. Solid lines: pure component isotherm fit using the Sips equation. Dashed lines: predictions using IAST.

S6. Determination of load-dependent heats of adsorption

To determine simultaneously adsorption isotherms and load-dependent heats of adsorption, a commercially available volumetric measuring device (autosorb iQ3, Quantachrome Instruments, Boynton Beach, FL, USA) was coupled with a sample vessel developed by Bläker et al. [10.1016/j.micromeso.2016.12.037, 10.1016/j.micromeso.2020.110205]. The sensor gas calorimeter is symmetrical constructed with a measuring cell and a reference cell (see figure S3). Both cells have an inner and an outer volume. The inner volumes of the cells are connected to each other and the volumetric measuring device. The adsorbent is located in the measuring cell while the reference cell is filled with inert glass beads. Adsorption only occurs in the measuring cell. The two outer volumes of the cells are filled with a sensor gas and connected to a pressure sensor. The entire sample vessel is placed in a water bath to limit external disturbances and to ensure a constant measuring temperature.

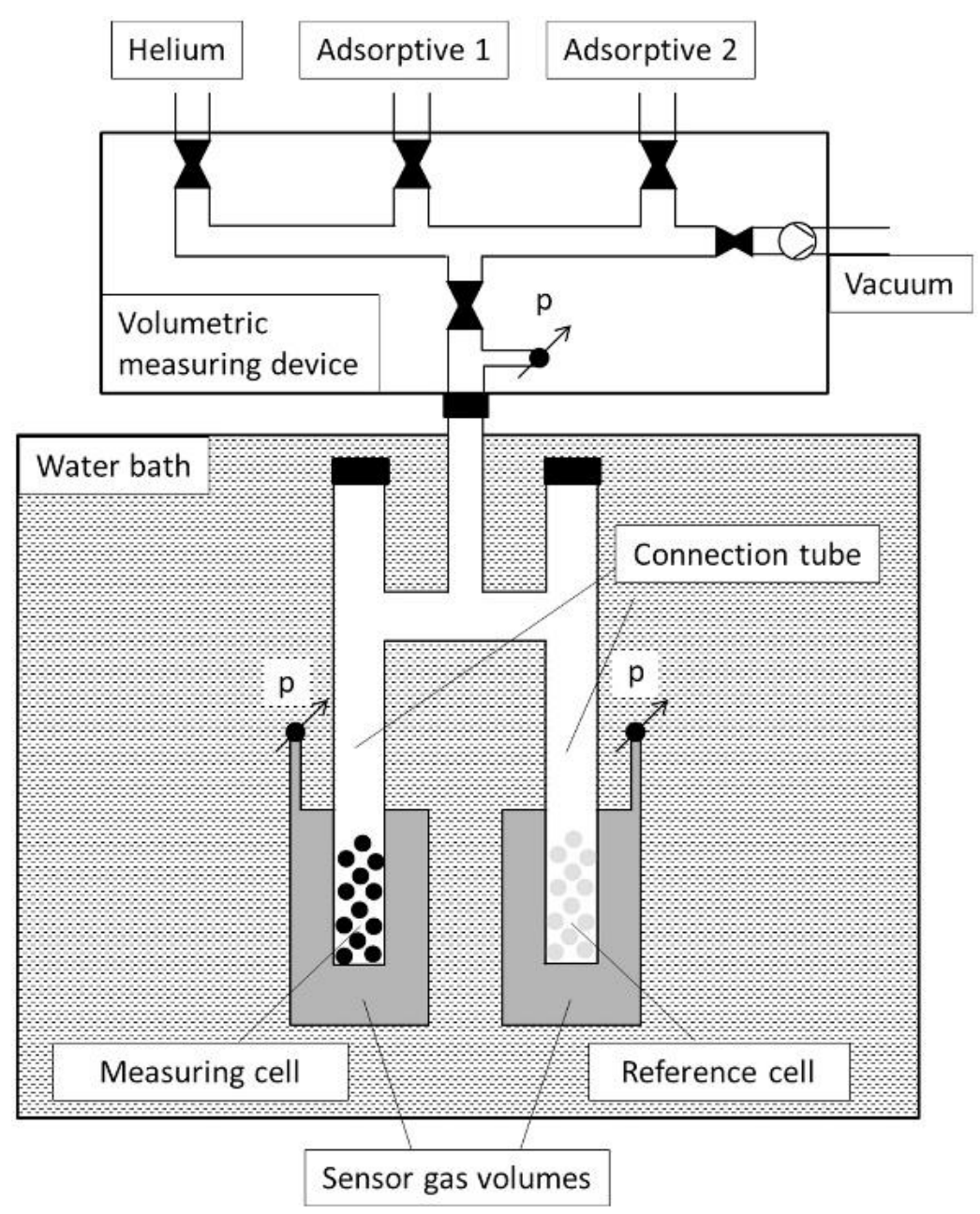

Figure S3: Schematic setup of the sensor gas calorimeter [10.1016/j.micromeso.2016.12.037].

Using the volumetric measuring instrument, pure component isotherms between high vacuum (10^{-3} Pa) and ambient pressure (100 kPa) can be measured cumulatively. During each equilibrium step, a specific amount of substance is dosed into the manifold. This is followed by pressure equalization between the manifold and the sample vessel, causing part of the substance to enter the sample

vessel and be adsorbed on the adsorbent. Once a constant pressure is established, equilibrium is reached. A pressure change of 0.3% over a period of 20 minutes is used as the equilibrium criterion. During an equilibrium step, the added amount of substance thus leads to an increase in loading and a rise in pressure. However, since a specific equilibrium pressure cannot be precisely set based solely on the dosed amount of substance, the step size depends on the system consisting of the adsorbent and the adsorptive as well as the amount of substance dosed. Consequently, it is not possible to specify a uniform adsorption step size. During the exothermic adsorption, heat is released in the measuring cell which results in a heat flow from the measuring cell through the surrounding sample gas volume into the water bath. This heat flow leads to a temperature and pressure change in the sensor gas volume of the measuring cell. In the reference cell, no adsorption on the inert glass beads takes place and therefore the temperature and pressure in this sensor gas volume are constant over the entire measuring period. Consequently, a time-dependent pressure difference between the two sensor gas volumes is measured. The pressure difference curve drops back to the baseline after each equilibrium step, so no baseline correction is necessary. For each adsorption step, the released heat of adsorption is calculated from the area under the resulting time-dependent difference curve A_{Peak}, the mass of adsorbent m_{Ads}, the loading difference Δn_a and a calibration factor Cf using equation S34. Consequently, the plotted values represent negative adsorption enthalpies.

$$\Delta h_{Ads,i} = -\frac{A_{Peak,i} \cdot Cf}{\Delta n_{a,i} \cdot m_{Ads}} \tag{S34}$$

Joule heating (also known as ohmic or resistive heating) is used to determine the device-specific calibration factor. Joule heating is the process where electrical energy is converted into heat as an electric current passes through a conductor. A defined amount of electrical energy flows through a conductor located in the measuring cell and is converted into heat, which is detected by the sensor gas. The calibration factor is based on the correlation shown in equation S35 between the area under the resulting pressure difference curve and the heating energy. The physical calibration factor acts as a proportionality constant and corresponds to the slope of the regression line.

$$Cf\,(T_{Mess}) = \frac{\Delta Q_{ads}(k)}{A_{Peak}(k)} = \frac{\Delta E_{el}(k)}{A_{Peak}(k)} \tag{S35}$$

Before the measurement, the zeolites are heated at 300°C for 6 hours, following the same procedure used in breakthrough experiments. To verify the measurement principle, repeat measurements were performed, and the measurement error was calculated using Gaussian error propagation. The deviations between the repeat measurements as well as the calculated measurement error are in the range of 1–2 kJ/mol.

Based on our many years of experience with the measurement setup and the plausible data on propane adsorption, which are comparable to those reported in literature, we did not perform duplicate measurements for any of the measurements shown in the manuscript.

S7. XRPD results

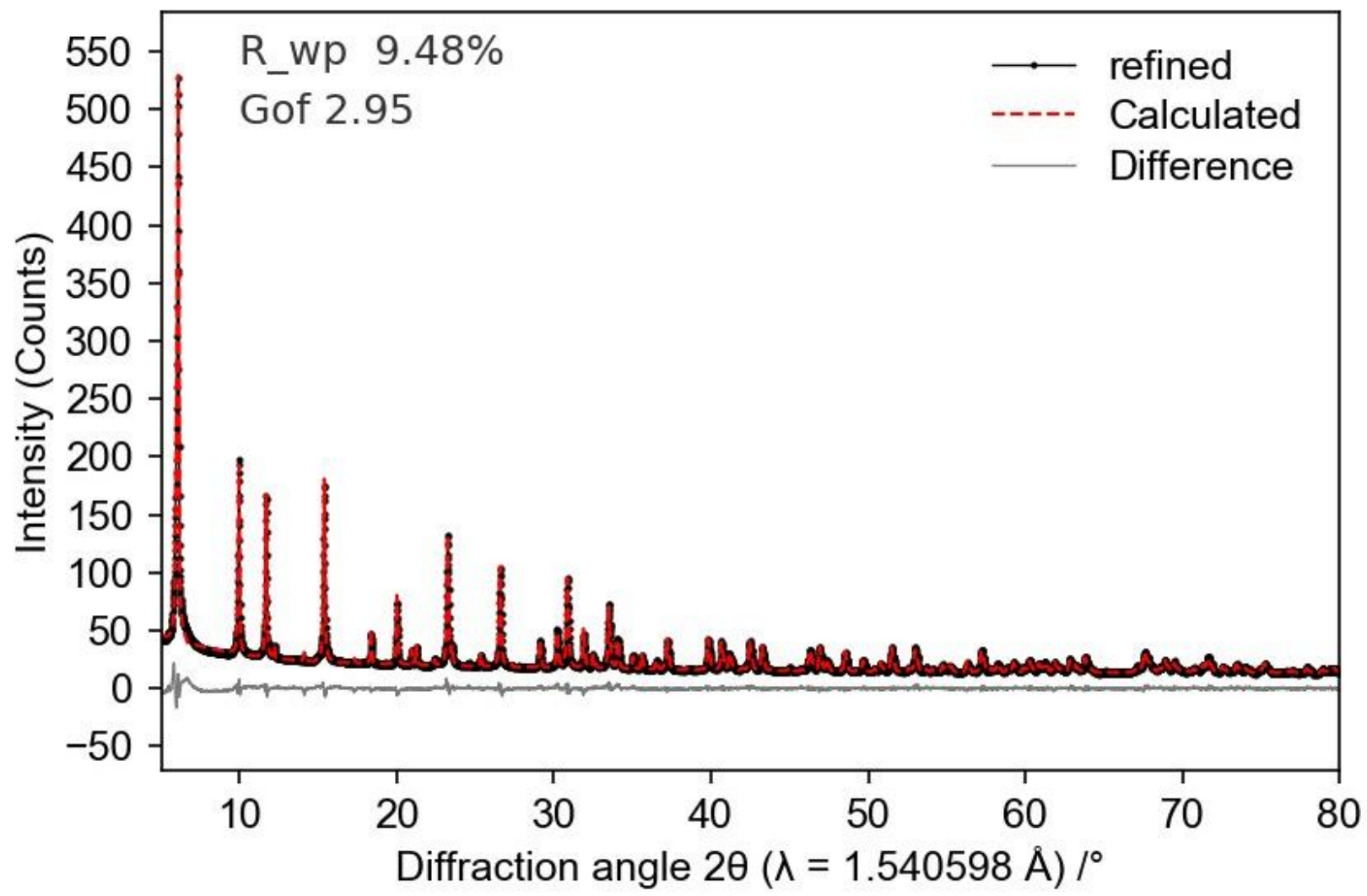

Figure S4: Final Rietveld refinement plot obtained for $Na_{88}Si_{104}Al_{88}O_{384}$. The measured XRPD data are displayed as solid lines, the calculated XRPD patterns from the refined models are shown as red dotted lines, the difference curves are shown as solid grey lines.

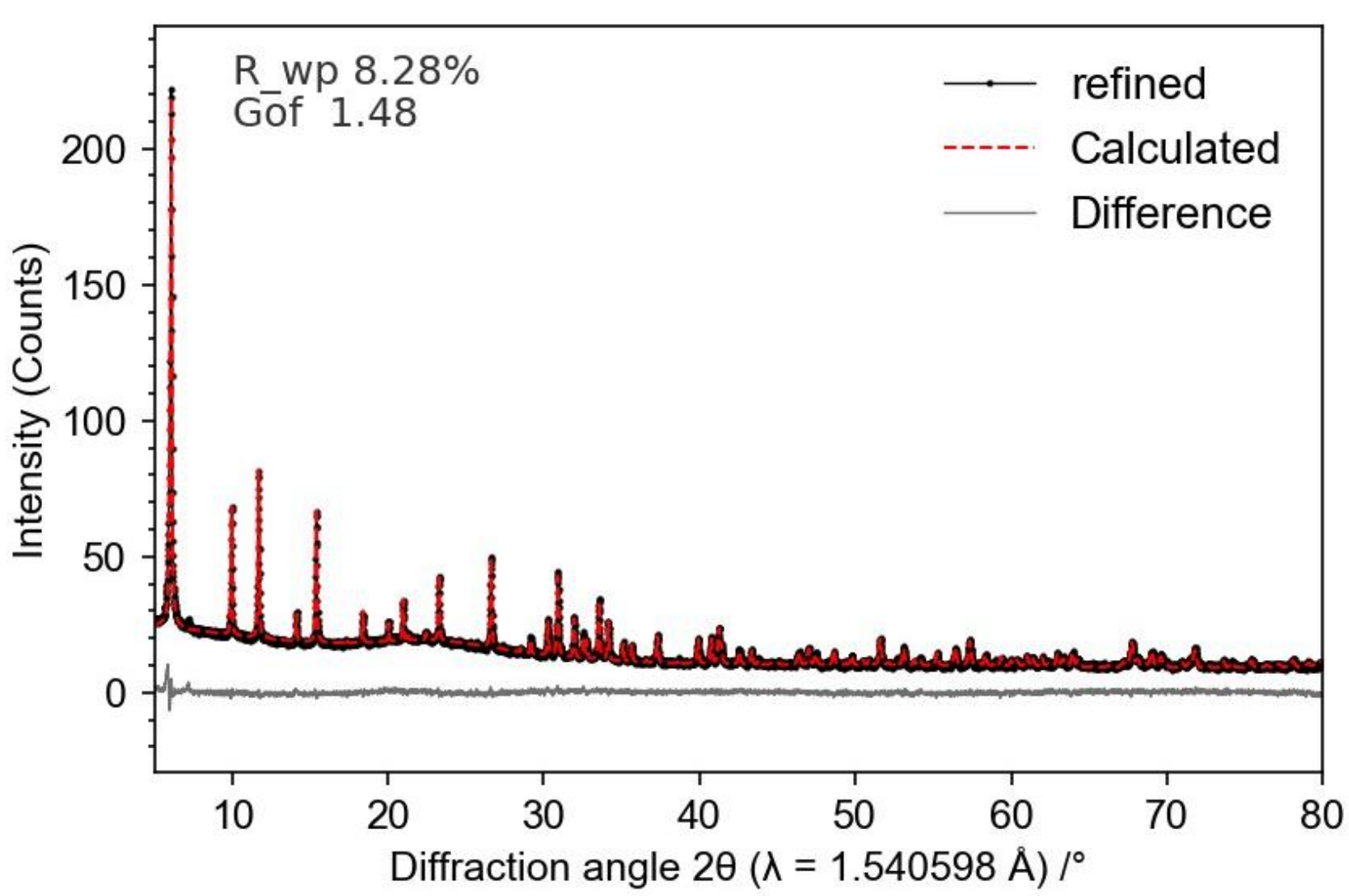

Figure S5: Final Rietveld refinement plot obtained for $Na_{16}Ca_{36}Si_{104}Al_{88}O_{384}$. The measured XRPD data are displayed as solid lines, the calculated XRPD patterns from the refined models are shown as red dotted lines, the difference curves are shown as solid grey lines.
